# Energy transfer of imbalanced Alfvénic turbulence in the heliosphere

**DOI:** 10.1038/s41467-023-43273-4

**Published:** 2023-12-02

**Authors:** Liping Yang, Jiansen He, Daniel Verscharen, Hui Li, Trevor A. Bowen, Stuart D. Bale, Honghong Wu, Wenya Li, Ying Wang, Lei Zhang, Xueshang Feng, Ziqi Wu

**Affiliations:** 1 SIGMA Weather Group, State Key Laboratory of Space Weather, National Space Science Center, Chinese Academy of Sciences, 100190 Beijing, People’s Republic of China; 2 School of Earth and Space Sciences, Peking University, 100871 Beijing, People’s Republic of China; 3 Mullard Space Science Laboratory, University College London, Surrey, RH5 6NT UK; 4 Theoretical Division, Los Alamos National Laboratory, Los Alamos, NM 87545 USA; 5 Space Sciences Laboratory, University of California, Berkeley, CA 94720-7450 USA; 6 Physics Department, University of California, Berkeley, CA 94720-7300 USA; 7 School of Electronic Information, Wuhan University, 430072 Wuhan, People’s Republic of China; 8 State Key Laboratory of Space Weather, National Space Science Center, Chinese Academy of Sciences, 100190 Beijing, People’s Republic of China; 9 China Academy of Aerospace Science and Innovation, 100190 Beijing, People’s Republic of China

**Keywords:** Space physics, Plasma physics, Magnetospheric physics

## Abstract

Imbalanced Alfvénic turbulence is a universal process playing a crucial role in energy transfer in space,  astrophysical, and laboratory plasmas. A fundamental and long-lasting question about the imbalanced Alfvénic turbulence is how and through which mechanism the energy transfers between scales. Here, we show that the energy transfer of imbalanced Alfvénic turbulence is completed by coherent interactions between Alfvén waves and co-propagating anomalous fluctuations. These anomalous fluctuations are generated by nonlinear couplings instead of linear reflection. We also reveal that the energy transfer of the waves and the anomalous fluctuations is carried out mainly through local-scale and large-scale nonlinear interactions, respectively, responsible for their bifurcated power-law spectra. This work unveils the energy transfer physics of imbalanced Alfvénic turbulence, and advances the understanding of imbalanced Alfvénic turbulence observed by Parker Solar Probe in the inner heliosphere.

## Abstract (web summary)

How and via which mechanism the energy transfers between scales in imbalanced Alfvénic turbulence is an open question. Here, the authors show that the energy transfer of imbalanced Alfvénic turbulence is completed by coherent interactions between Alfvén waves and co-propagating anomalous fluctuations.

## Introduction

Magnetohydrodynamic (MHD) turbulence is believed to play key roles in a wide range of plasma environments, e.g., astrophysical systems, the solar system, and plasma laboratories. The collision between counter-propagating Alfvén wave packets is credited as the fundamental physical process that drives the energy cascade of MHD turbulence. This framework has become the predominant phenomenological description in theoretical studies of MHD turbulence^1–4^. During such a wave collision, a wave packet suffers a nonlinear distortion from its interaction with the encountered oppositely traveling wave packet. When the wave packet undergoes an order-unity distortion, its energy is deemed to have cascaded to a smaller scale^5^. MHD turbulence is called “imbalanced” if the power of counter-propagating Alfvén waves is different in both directions with respect to the background magnetic field direction^2–10^. MHD turbulence is classified into weak and strong turbulence according to the strength of nonlinear interactions compared to linear interactions^3,11,12^. Nonlinear processes and the corresponding energy spectrum of the MHD turbulence are still among the most controversial problems in MHD research^13^. It is especially still unclear whether strong nonlinear distortions make the waves lose their identity, or whether nonlinear interactions between waves continue in a wave-like sense along the energy cascade after multiple encounters.

Another important concern in MHD turbulence theory is the scale locality of the cascade, which is embedded in the classical theory of hydrodynamic turbulence and leads to the well-known Kolmogorov turbulent scaling laws^13^. Scale locality means that a fluctuation transfers energy to smaller-scale fluctuations primarily through the interactions with fluctuations of similar size or similar wavenumber in Fourier space^14^. There is a hot debate about the scale locality in MHD turbulence since large-scale magnetic fields cannot be removed through a Galilean transformation^13,15–18^.

As a magnetized super-sonic plasma flow emanating from the Sun and filling the heliosphere, the solar wind is found to be turbulent according to in-situ measurements from space missions. Solar wind turbulence is usually imbalanced, especially close to the Sun^19–23^. The turbulence supplies energy to heat and accelerates the solar wind plasma, especially in the solar corona and the inner heliosphere. The turbulent solar wind serves as the medium in which energetic particles travel and are scattered. It thus has a critical influence on the dynamics and kinetics of the heliosphere. Therefore, studying solar wind turbulence is necessary to understand the transport and transfer of energy and momentum, solar–terrestrial relations, as well as fundamental plasma physics.

One of the cutting-edge problems about solar wind turbulence is how the energy transfers through nonlinear interactions across scales and ultimately heats the solar wind. Elsässer variables ${{{{{{\rm{\delta }}}}}}{{{{{\bf{Z}}}}}}}^{\pm }$ describe two eigenmodes of Alfvén waves propagating in opposite directions^24^, and nonlinear interactions between ${{{{{{\rm{\delta }}}}}}{{{{{\bf{Z}}}}}}}^{\pm }$ generate energy cascade of the solar wind turbulence^25–28^. To sustain the subdominant inward propagating Alfvén waves, it is necessary to introduce the reflection of the dominant outward propagating Alfvén waves or a local source, such as velocity shears or plasma instabilities. However, the cascade due to the collision between counter-propagating waves is challenged by the fact that the solar wind fluctuations initially consist of almost purely outward propagating Alfvén waves near the Sun. In addition, this cascade scenario faces challenges in comparison to the observed spectral behavior of the solar wind turbulence^23,29,30^.

In this work, we use Parker Solar Probe (PSP) observations and numerical simulations to examine the two foundational pillars of imbalanced Alfvénic turbulence: the nature of fluctuations involved in nonlinear interactions and the scale locality of the cascade. We propose the following scenario: the outward Alfvén waves (${{{{{{\rm{\delta }}}}}}{{{{{\bf{Z}}}}}}}^{+}$) no longer interact with inward Alfvén waves, but instead interact with co-propagating fluctuations (${{{{{{\rm{\delta }}}}}}{{{{{\bf{Z}}}}}}}^{-}$), to produce an energy cascade. Through both local-scale and large-scale interactions, such a cascade process describes well both the observed and simulated imbalanced Alfvénic turbulence. Our findings unveil nonlinear interactions in imbalanced Alfvénic turbulence and lay the groundwork for the theoretical description of the imbalanced Alfvénic turbulent cascade, which can be fed into dynamical models.

## Results

### Alfvénic turbulence observed by the PSP spacecraft

**Fig. 1:**
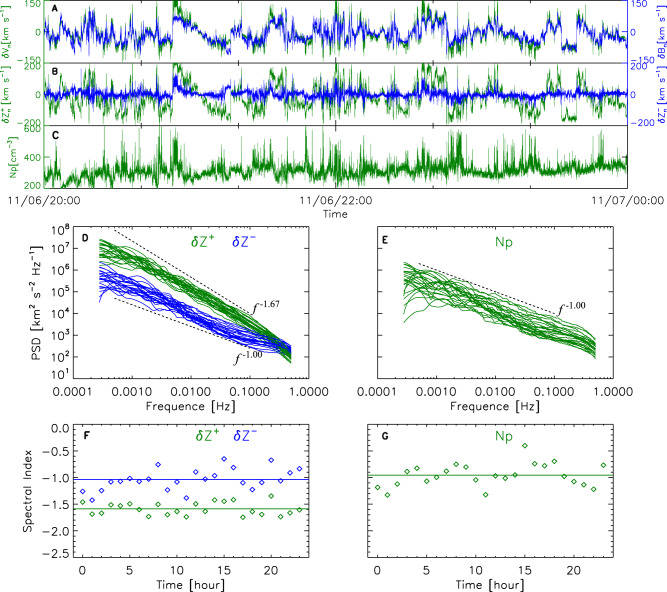
Alfvénic turbulence observed by PSP. **A**–**C** Time series of the fluctuating variables: plasma velocity component ${{{{{\rm{\delta }}}}}}{{{{{{\rm{V}}}}}}}_{n}$ (**A**, green line), magnetic field component ${{{{{\rm{\delta }}}}}}{{{{{{\rm{B}}}}}}}_{n}$ (**A**, blue line), Elsässer components ${{{{{\rm{\delta }}}}}}{{{{{{\bf{Z}}}}}}}^{+}$ (**B**, green line) and ${{{{{\rm{\delta }}}}}}{{{{{{\bf{Z}}}}}}}^{-}$ (**B**, blue line), and proton number density ${N}_{\rm {{p}}}$ (**C**, green line). **D** and **E** variation of power spectral density (PSD) of ${{{{{\rm{\delta }}}}}}{{{{{{\bf{Z}}}}}}}^{+}$ (**D**, green lines), ${{{{{\rm{\delta }}}}}}{{{{{{\bf{Z}}}}}}}^{-}$ (**D**, blue lines), and the proton number density ${N}_{\rm {{p}}}$ (**E**, green lines) with frequency $f$ on one-hour intervals. Two power-law slopes are marked in black for reference. **F** and **G** The variations of the power-law spectral indexes with time, with their means marked by the horizontal lines.

Our observational data is from the PSP spacecraft, which approaches the Sun to explore the nature of MHD turbulence in the new frontier of the young solar wind^31^. Figure 1 shows measurements by the PSP at its perihelion distance of 0.17 a.u. on 6 November 2018. The perturbed plasma velocity ${{{{{\rm{\delta }}}}}}{{{{{\bf{V}}}}}}$ and magnetic field ${{{{{\rm{\delta }}}}}}{{{{{\bf{B}}}}}}$ display an excellent correlation. The normalized cross-helicity ${\sigma }_{{\rm {c}}}$ is 0.84. The angle between the sampling direction and the ambient magnetic field direction is about ${132}^{^\circ }$.

Figure 1 also shows that the amplitude of the fluctuating Elsässer variable ${{{{{{\rm{\delta }}}}}}{{{{{\bf{Z}}}}}}}^{+}$ is generally much greater than the amplitude of its counterpart ${{{{{\rm{\delta }}}}}}{{{{{{\bf{Z}}}}}}}^{-}$, indicating the presence of imbalanced Alfvénic turbulence. The trace power spectra of ${{{{{\rm{\delta }}}}}}{{{{{{\bf{Z}}}}}}}^{+}$ are statistically steeper than the spectra of ${{{{{\rm{\delta }}}}}}{{{{{{\bf{Z}}}}}}}^{-}$ (Fig. 1D). Such spectral differences in a short frequency range have already been observed by Helios^32^, although some cases occasionally show similar spectral steepness^33^. The power-law spectral index of ${{{{{\rm{\delta }}}}}}{{{{{{\bf{Z}}}}}}}^{+}$ is around −1.6, while the spectral indexes of ${{{{{\rm{\delta }}}}}}{{{{{{\bf{Z}}}}}}}^{-}$ and the proton number density ${N}_{\rm {{p}}}$ are around −1.0.

The average spectral slopes of ${{{{{\rm{\delta }}}}}}{{{{{{\bf{Z}}}}}}}^{+}$(-1.6) and ${{{{{\rm{\delta }}}}}}{{{{{{\bf{Z}}}}}}}^{-}$ (-1.0) are features of the solar wind in the frequency range between 0.001 and 0.1 Hz on 6 November 2018. Shi et al.^34^ show that, at a mean cross-helicity of 0.9, the typical spectral slopes are -1.64 (${{{{{\rm{\delta }}}}}}{{{{{{\bf{Z}}}}}}}^{+}$) and -0.99 (${{{{{\rm{\delta }}}}}}{{{{{{\bf{Z}}}}}}}^{-}$), and at a mean cross-helicity of 0.93, they are -1.55 (${{{{{\rm{\delta }}}}}}{{{{{{\bf{Z}}}}}}}^{+}$) and -1.10 (${{{{{\rm{\delta }}}}}}{{{{{{\bf{Z}}}}}}}^{-}$). This comparison suggests that our average spectral slopes are representative of the PSP data with very high cross-helicity. There is also precedence for a spectral slope of −1 in the high-frequency inertial range for the density power spectrum in the solar wind in Helios data, where Marsch and Tu^35^ report a flattening of the density power spectrum. Bruno et al.^36^ report that most of the flattening is due to intermittent events and the spectral slopes are all approximately −1. Borovsky et al.^37^ find a spectral index of the number density spectrum at high frequencies of -0.58 ± 0.36 in ACE data.

### Alfvénic turbulence reproduced by numerical simulation

To investigate the physics of Alfvénic turbulence in the heliosphere, we conduct a decaying turbulence simulation by advancing the dimensionless compressible MHD equations in a periodic cube with 1024^3^ grid points^38,39^. Considering that it is unclear what realistic driving would look like and additional dependence on the driver may be introduced into the system, here we use a decaying turbulence setup^28,40^ instead of a setup with driven turbulence. At a constant heliocentric distance, the turbulence in the solar wind is considered statistically stationary. When considering the radial evolution of the solar wind turbulence, however, its energy shows a decreasing profile with heliocentric distance^41^. The decrease is not only due to the solar wind expansion but also the decay of turbulence^19^.

The initial fluctuation energy is equipartitioned between the kinetic and magnetic components, and the initial fluctuations are defined as Alfvén waves. The initial normalized cross-helicity ${{{{{{\rm{\sigma }}}}}}}_{{{{{{\rm{c}}}}}}}$ is set to be approximately 0.7. In general, cross-helicity can be expressed in terms of ${{{{{\rm{\delta }}}}}}{{{{{\bf{V}}}}}}$ and ${{{{{\rm{\delta }}}}}}{{{{{\bf{B}}}}}}$ as $2 < {{{{{\rm{\delta }}}}}}{{{{{\bf{V}}}}}}\cdot {{{{{\rm{\delta }}}}}}{{{{{\bf{B}}}}}} > /( < {{{{{{\rm{|}}}}}}{{{{{\rm{\delta }}}}}}{{{{{\bf{V}}}}}}{{{{{\rm{|}}}}}}}^{{{{{{\bf{2}}}}}}} > + < {{{{{{\rm{|}}}}}}{{{{{\rm{\delta }}}}}}{{{{{\bf{B}}}}}}{{{{{\rm{|}}}}}}}^{{{{{{\bf{2}}}}}}} > )$, and in terms of ${{{{{{\rm{\delta }}}}}}{{{{{\bf{Z}}}}}}}^{+}$ and ${{{{{{\rm{\delta }}}}}}{{{{{\bf{Z}}}}}}}^{-}$ as $( < {{{{{{{\rm{|}}}}}}{{{{{\rm{\delta }}}}}}{{{{{\bf{Z}}}}}}}^{+}{{{{{\rm{|}}}}}}}^{2} > - < {{{{{{{\rm{|}}}}}}{{{{{\rm{\delta }}}}}}{{{{{\bf{Z}}}}}}}^{-}{{{{{\rm{|}}}}}}}^{2} > )/( < {{{{{{{\rm{|}}}}}}{{{{{\rm{\delta }}}}}}{{{{{\bf{Z}}}}}}}^{+}{{{{{\rm{|}}}}}}}^{2} > + < {{{{{{{\rm{|}}}}}}{{{{{\rm{\delta }}}}}}{{{{{\bf{Z}}}}}}}^{-}{{{{{\rm{|}}}}}}}^{2} > )$. By adjusting the relative amplitudes of ${{{{{{\rm{\delta }}}}}}{{{{{\bf{Z}}}}}}}^{+}$ and ${{{{{{\rm{\delta }}}}}}{{{{{\bf{Z}}}}}}}^{-}$, we set the initial normalized cross-helicity. Furthermore, we utilize the relationships between ${{{{{\rm{\delta }}}}}}{{{{{\bf{V}}}}}}$ and $({{{{{{\rm{\delta }}}}}}{{{{{\bf{Z}}}}}}}^{+}+{{{{{{\rm{\delta }}}}}}{{{{{\bf{Z}}}}}}}^{-})/2$ as well as ${{{{{\rm{\delta }}}}}}{{{{{\bf{B}}}}}}$ and $({{{{{{\rm{\delta }}}}}}{{{{{\bf{Z}}}}}}}^{+}-{{{{{{\rm{\delta }}}}}}{{{{{\bf{Z}}}}}}}^{-})/2$, to obtain the initial spatial distributions of ${{{{{\rm{\delta }}}}}}{{{{{\bf{V}}}}}}$ and ${{{{{\rm{\delta }}}}}}{{{{{\bf{B}}}}}}$.

**Fig. 2:**
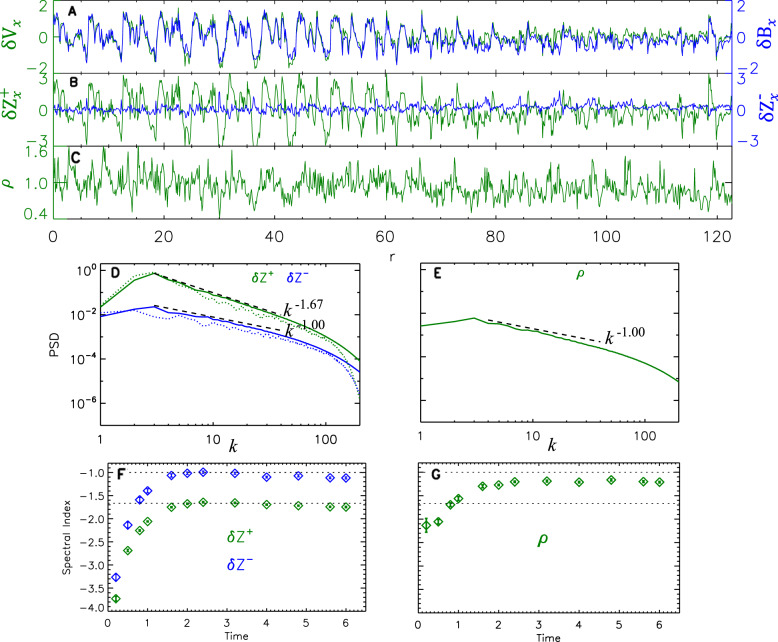
Alfvénic turbulence sampled in the numerical simulation data. **A**–**C** Spatial series of the fluctuating variables: velocity component ${{{{{\rm{\delta }}}}}}{{{{{{\rm{V}}}}}}}_{x}$ (**A**, green line), magnetic field component ${{{{{\rm{\delta }}}}}}{{{{{{\rm{B}}}}}}}_{x}$ (**A**, blue line), Elsässer components ${{{{{\rm{\delta }}}}}}{{{{{{\rm{Z}}}}}}}_{x}^{+}$ (**B**, green line) and ${{{{{\rm{\delta }}}}}}{{{{{{\rm{Z}}}}}}}_{x}^{-}$ (**B**, blue line), and density ${{{{{\rm{\rho }}}}}}$ (**C**, green line) at ${{{{{\rm{time}}}}}}=4.0$. **D** and **E** variation of power spectral density (PSD) of ${{{{{\rm{\delta }}}}}}{{{{{{\bf{Z}}}}}}}^{+}$ (**D**, green lines), ${{{{{\rm{\delta }}}}}}{{{{{{\bf{Z}}}}}}}^{-}$ (**D**, blue lines), and the density ${{{{{\rm{\rho }}}}}}$ (**E**, green lines) with wavenumber $k$. The dotted lines in **D** show the power spectra of ${{{{{\rm{\delta }}}}}}{{{{{{\bf{Z}}}}}}}^{+}$ and ${{{{{\rm{\delta }}}}}}{{{{{{\bf{Z}}}}}}}^{-}$ at ${{{{{\rm{time}}}}}}=4.0$ from our imbalanced RMHD simulation. Two power-law slopes are marked in black for reference. **F** and **G** The evolution of the power-law spectral indexes as functions of the simulation time. The error bars denote uncertainties of the linear fit on log–log scales. The same ranges on the vertical axes are adopted for (**D** and **E**) as well as (**F** and **G**). The displayed variables are dimensionless.

Figure 2 displays the features of our simulated MHD turbulence, which we sample with a virtual satellite whose trajectory resembles PSP’s orbital geometry in our observations. The periodic boundary conditions allow the virtual satellite to pass through and reenter the simulation domain many times. This approach provides the spacecraft trajectory with about 10^5^ sampling points. The spectra, calculated by fast Fourier transform of the sampled points, remain constant when we sample for more than 10^5^ points. Figure 2 shows that, at ${{{{{\rm{time}}}}}}=4.0$, the transverse component of ${{{{{\rm{\delta }}}}}}{{{{{\bf{V}}}}}}$ is well correlated with the transverse component of ${{{{{\rm{\delta }}}}}}{{{{{\bf{B}}}}}}$, leading to ${\sigma }_{\rm {{c}}}$ ~ 0.78. The simulated ${{{{{\rm{\delta }}}}}}{{{{{{\bf{Z}}}}}}}^{+}$ follows a Kolmogorov (−5/3) spectrum, while the simulated ${{{{{\rm{\delta }}}}}}{{{{{{\bf{Z}}}}}}}^{-}$ preserves a power-law spectral index of about −1. Furthermore, the spectrum of density $\rho$ stabilizes at a power-law index close to −1 as well. The simulated density fluctuations have relative amplitudes of about 20% of the mean density, which is slightly greater than those of the solar wind density fluctuations in Fig. 1 (about 15%).

The simulation reproduces well the features of the Alfvénic fluctuations, which are observed in certain intervals of PSP measurements. This can also be seen in Supplementary Fig. 1, which shows the similarity between the simulated and observed distributions of occurrence numbers and pixel-averaged ${ < {{{{{\rm{|}}}}}}{{{{{\rm{\delta }}}}}}{{{{{{\bf{V}}}}}}}_{{{{{{\rm{A}}}}}}}^{2}{{{{{\rm{|}}}}}} > }^{0.5}$ in the ${\sigma }_{{\rm {r}}}-{\sigma }_{{\rm {c}}}$ plane, as well as variations of the intermittency-related scaling exponents with the order moments of the structure function.

Supplementary Fig. 2 shows 2D representations of the discretized differential variables (${{{{{\rm{|}}}}}}{{{{{\boldsymbol{\nabla }}}}}}\times {{{{{\bf{V}}}}}}{{{{{\rm{|}}}}}}$, ${{{{{\rm{|}}}}}}{{{{{\boldsymbol{\nabla }}}}}}\cdot {{{{{\bf{V}}}}}}{{{{{\rm{|}}}}}}$, $\partial \rho /\partial t$, and ${{{{{\bf{V}}}}}}\cdot {{{{{\boldsymbol{\nabla }}}}}}\rho$), the magnetic pressure ${P}_{{{{{{\rm{mag}}}}}}}$, and the thermal pressure ${P}_{{{{{{\rm{th}}}}}}}$. The curvature of the velocity ${{{{{\rm{|}}}}}}{{{{{\boldsymbol{\nabla }}}}}}\times {{{{{\bf{V}}}}}}{{{{{\rm{|}}}}}}$ is far greater than the divergence of the velocity ${{{{{\rm{|}}}}}}{{{{{\boldsymbol{\nabla }}}}}}\cdot {{{{{\bf{V}}}}}}{{{{{\rm{|}}}}}}$. The distribution of $\left|{{{{{\boldsymbol{\nabla }}}}}}\cdot {{{{{\bf{V}}}}}}\right|$ seems irregular and noise-like. The time derivative $\partial \rho /\partial t$ nearly equates to ${{{{{\bf{V}}}}}}\cdot {{{{{\boldsymbol{\nabla }}}}}}\rho$ (see the region marked by the white rectangles), suggesting that the advection dominates over the compressibility to generate the variation of density. Finally, the magnetic pressure ${P}_{{{{{{\rm{mag}}}}}}}$ and the thermal pressure ${P}_{{{{{{\rm{th}}}}}}}$ are in good anti-correlation (see the regions marked by the black rectangles), forming multiscale pressure-balanced structures (PBSs), which have also been observed in solar wind turbulence before^42,43^. The balance between the magnetic pressure and the thermal pressure weakens the role of ponderomotive force related to the spatial variation of magnetic field magnitude in generating more compressible fluctuations. Therefore, with a highly Alfvénic, incompressible initial condition, our compressible MHD simulation converges to a weakly compressible state. A comparison of Figs. 2, S1, and S2 shows that the converged status with both high Alfvénicity and weak compressibility resembles the solar wind turbulence measured by the PSP.

### Nature of fluctuations involved in nonlinear interactions

**Fig. 3:**
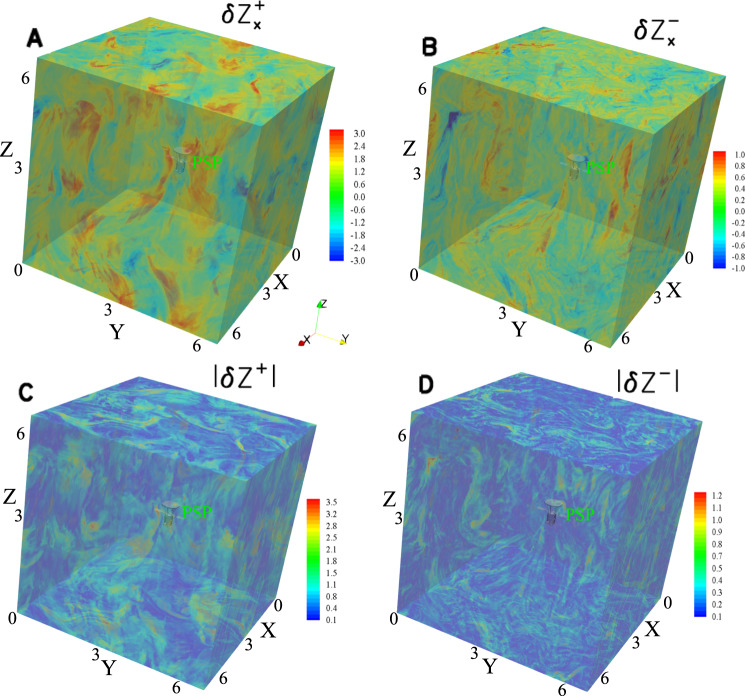
Distributions of the turbulent Elsässer variables in the simulation domain at time = 4.0. **A** and **B** The perpendicular components of Elsässer variables ${{{{{\rm{\delta }}}}}}{{{{{{\rm{Z}}}}}}}_{x}^{+}$ and ${{{{{\rm{\delta }}}}}}{{{{{{\rm{Z}}}}}}}_{x}^{-}$. **C** and **D** The magnitudes of ${{{{{\rm{|}}}}}}{{{{{\rm{\delta }}}}}}{{{{{{\bf{Z}}}}}}}^{+}{{{{{\rm{|}}}}}}$ and ${{{{{\rm{|}}}}}}{{{{{\rm{\delta }}}}}}{{{{{{\bf{Z}}}}}}}^{-}{{{{{\rm{|}}}}}}$. The displayed variables are dimensionless. The model of the PSP is illustrated in green at the center of the domain. The simulation box and the spacecraft model are not scaled to size. Their corresponding temporal propagation and evolution are shown in Supplementary Movie 1.

We illustrate the spatio-temporal behavior of the simulated $x{{{{{\boldsymbol{-}}}}}}$ component of the Elsässer variables, ${{{{{\rm{\delta }}}}}}{{{{{{\rm{Z}}}}}}}_{x}^{\pm }$, and ${{{{{\rm{|}}}}}}{{{{{\rm{\delta }}}}}}{{{{{{\bf{Z}}}}}}}^{\pm }{{{{{\rm{|}}}}}}$ for the time from 4.0 until 6.0 in Fig. 3 and Supplementary Movie 1. The fluctuations associated with ${{{{{\rm{\delta }}}}}}{{{{{{\bf{Z}}}}}}}^{+}$ travel in the direction opposite to the guide field ${{{{{{\bf{B}}}}}}}_{0}$, in agreement with the dispersion relation for anti-parallel propagating Alfvén waves. However, the fluctuations associated with the minor Elsässer variable ${{{{{\rm{\delta }}}}}}{{{{{{\bf{Z}}}}}}}^{-}$ do not counter-propagate with respect to ${{{{{\rm{\delta }}}}}}{{{{{{\bf{Z}}}}}}}^{+}$. Instead, the fluctuations of ${{{{{\rm{\delta }}}}}}{{{{{{\bf{Z}}}}}}}^{-}$ propagate in the same direction as ${{{{{\rm{\delta }}}}}}{{{{{{\bf{Z}}}}}}}^{+}$.

**Fig. 4:**
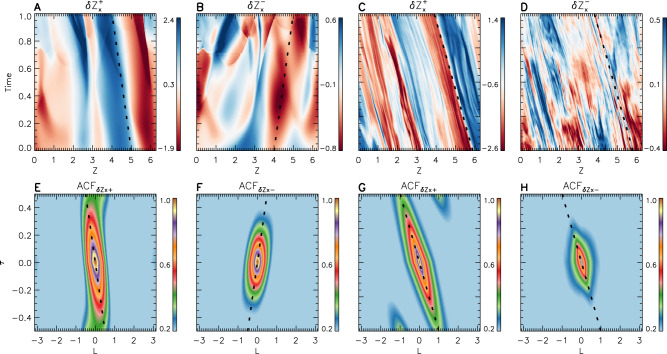
Propagation and lifetime estimations of $\delta {Z}_{x}^{+}$ and $\delta {Z}_{x}^{-}$. **A**–**D** Time-distance (${{{{{\rm{time}}}}}}-z$) diagrams of ${{{{{\rm{\delta }}}}}}{{{{{{\rm{Z}}}}}}}_{x}^{+}$ and ${{{{{\rm{\delta }}}}}}{{{{{{\rm{Z}}}}}}}_{x}^{-}$ for the time from 0.0 until 1.0 (**A** and **B**) and from 4.0 until 6.0 (**C** and **D**). **E**–**H** Distributions of the auto-correlation function (ACF) of ${{{{{\rm{\delta }}}}}}{{{{{{\rm{Z}}}}}}}_{x}^{+}$ and ${{{{{\rm{\delta }}}}}}{{{{{{\rm{Z}}}}}}}_{x}^{-}$ in the $\tau$ (time lag) $-l$ (spatial lag in the *z*-direction) plane for times between 0.0 and 1.0 (**E** and **F**) and for times between 4.0 and 6.0 (**G** and **H**). The black dashed lines show the expected propagation of Alfvén waves along the *z-*direction. The displayed variables are dimensionless.

We investigate the propagation speed of ${{{{{\rm{\delta }}}}}}{{{{{{\rm{Z}}}}}}}_{x}^{\pm }$ by extracting a 1D cut along the direction of the mean background field ${{{{{{\bf{B}}}}}}}_{0}$ (*z*-direction). This analysis produces the time-distance diagrams shown in Fig. 4A, B for the time from 0.0 until 1.0 and Fig. 4C, D for the time from 4.0 until 6.0. The diagrams for both ${{{{{\rm{\delta }}}}}}{{{{{{\rm{Z}}}}}}}_{x}^{+}$ and ${{{{{\rm{\delta }}}}}}{{{{{{\rm{Z}}}}}}}_{x}^{-}$ display oblique stripes, the slopes of which correspond to the propagation speed of ${{{{{\rm{\delta }}}}}}{{{{{{\rm{Z}}}}}}}_{{{{{{\rm{x}}}}}}}^{\pm }$ along the *z*-direction. Figure 4A and B show that ${{{{{\rm{\delta }}}}}}{{{{{{\rm{Z}}}}}}}_{x}^{+}$ and ${{{{{\rm{\delta }}}}}}{{{{{{\rm{Z}}}}}}}_{x}^{-}$ initially have different propagation speeds. For ${{{{{\rm{\delta }}}}}}{{{{{{\rm{Z}}}}}}}_{x}^{+}$, the propagation speed approaches ${-V}_{{{{{{\rm{A}}}}}}0}$, while for ${{{{{\rm{\delta }}}}}}{{{{{{\rm{Z}}}}}}}_{x}^{-}$, it is approximately ${V}_{{{{{{\rm{A}}}}}}0}$. However, for the time from 4.0 until 6.0 shown in Fig. 4C and D, the propagation speed of ${{{{{\rm{\delta }}}}}}{{{{{{\rm{Z}}}}}}}_{x}^{-}$ becomes comparable to that of ${{{{{\rm{\delta }}}}}}{{{{{{\rm{Z}}}}}}}_{x}^{+}$, which is about ${-V}_{{{{{{\rm{A}}}}}}0}$. This behavior reveals that ${{{{{{\rm{\delta }}}}}}{{{{{\bf{Z}}}}}}}^{-}$ is strongly distorted by ${{{{{{\rm{\delta }}}}}}{{{{{\bf{Z}}}}}}}^{+}$ after their encounters. Furthermore, the stripes of ${{{{{\rm{\delta }}}}}}{{{{{{\rm{Z}}}}}}}_{x}^{-}$ appear to be shorter-lived in time compared to those of ${{{{{\rm{\delta }}}}}}{{{{{{\rm{Z}}}}}}}_{x}^{+}$.

From the extracted values of ${{{{{\rm{\delta }}}}}}{{{{{{\rm{Z}}}}}}}_{x}^{\pm }$, we calculate the auto-correlation functions (ACFs) of ${{{{{\rm{\delta }}}}}}{{{{{{\rm{Z}}}}}}}_{x}^{\pm }$, which are functions of the spatial lag *l* and time lag $\tau$. We choose about 40 1D cuts to calculate the average ACF, and show their distributions in $\tau -l$ space in Fig. 4E and F for times between 0.0 and 1.0 and in Fig. 4G and H for times in between 4.0 and 6.0. For the times between 0.0 and 1.0, the high-level ACFs of ${{{{{\rm{\delta }}}}}}{{{{{{\rm{Z}}}}}}}_{x}^{+}$ and ${{{{{\rm{\delta }}}}}}{{{{{{\rm{Z}}}}}}}_{x}^{-}$ mainly concentrate around the line $l=-{V}_{{{{{{\rm{A}}}}}}0}\tau$ and the line $l={V}_{{{{{{\rm{A}}}}}}0}\tau$, respectively, confirming again that ${{{{{{\rm{\delta }}}}}}{{{{{\rm{Z}}}}}}}_{x}^{+}$ and ${{{{{\rm{\delta }}}}}}{{{{{{\rm{Z}}}}}}}_{x}^{-}$ are Alfvén fluctuations propagating in opposite directions. However, for times between 4.0 and 6.0, the high-level ACFs of ${{{{{\rm{\delta }}}}}}{{{{{{\rm{Z}}}}}}}_{x}^{+}$ and ${{{{{\rm{\delta }}}}}}{{{{{{\rm{Z}}}}}}}_{x}^{-}$ both concentrate around the line $l=-{V}_{{{{{{\rm{A}}}}}}0}\tau$ confirming that both ${{{{{\rm{\delta }}}}}}{{{{{{\rm{Z}}}}}}}_{x}^{+}$ and ${{{{{\rm{\delta }}}}}}{{{{{{\rm{Z}}}}}}}_{x}^{-}$ propagate in the same direction at the negative Alfvén speed. The auto-correlation time scales, which are estimated as the time lag associated with ${{{{{\rm{ACF}}}}}}={{{{{{\rm{e}}}}}}}^{-1}$, also indicate the rapidly evolving nature of ${{{{{\rm{\delta }}}}}}{{{{{{\rm{Z}}}}}}}_{x}^{-}$.

There is no evident propagation of ${{{{{\rm{\delta }}}}}}{{{{{{\rm{Z}}}}}}}_{x}^{\pm }$ along perpendicular directions (see Supplementary Fig. 4), meaning that ${{{{{\rm{\delta }}}}}}{{{{{{\rm{Z}}}}}}}_{x}^{\pm }$ travels anisotropically and mainly along the parallel direction. From the comparison between Fig. 4 and Supplementary Fig. 4, we find that the variations of the fluctuations along the perpendicular directions are more variable than those along the parallel direction. In balanced MHD turbulence with a value of ${ < {{{{{\rm{|}}}}}}{{{{{\rm{\delta }}}}}}{{{{{{\rm{B}}}}}}}_{x}^{2}{{{{{\rm{|}}}}}} > }^{0.5}/{B}_{0}$ comparable to that in our imbalanced MHD turbulence, counter-propagating waves persist throughout the development phase of the turbulence (see Supplementary Fig. 5).

To delve deeper into the nature and evolution of ${{{{{\rm{\delta }}}}}}{{{{{{\bf{Z}}}}}}}_{\perp }^{-}$, we employ a first-principles analysis. This involves comparing the spatiotemporal distribution and evolution of each term in the governing equations. After defining the generalized compressible term

1 $${T}_{{{{{{\rm{comp}}}}}}}=-\frac{1}{\rho }{{{{{\boldsymbol{\nabla }}}}}}{P}_{\rm {{t}}}+\frac{{{{{{\rm{\delta }}}}}}{{{{{{\bf{Z}}}}}}}_{\perp }^{+}-{{{{{\rm{\delta }}}}}}{{{{{{\bf{Z}}}}}}}_{\perp }^{-}}{8}{{{{{\boldsymbol{\nabla }}}}}}\cdot \left(3{{{{{{\rm{\delta }}}}}}{{{{{\bf{Z}}}}}}}^{-}-{{{{{{\rm{\delta }}}}}}{{{{{\bf{Z}}}}}}}^{+}\right)-\frac{{{{{{\rm{\delta }}}}}}{{{{{{\bf{Z}}}}}}}_{\perp }^{+}-{{{{{\rm{\delta }}}}}}{{{{{{\bf{Z}}}}}}}_{\perp }^{-}}{2}{{{{{\boldsymbol{\nabla }}}}}}\cdot {{{{{{\bf{V}}}}}}}_{{{{{{\rm{A}}}}}}0}$$

with ${P}_{\rm {{t}}}$ being the total pressure, the compressible equation of the perpendicular fluctuating Elsässer variable ${{{{{\rm{\delta }}}}}}{{{{{{\bf{Z}}}}}}}_{\perp }^{-}$ (see Supplementary Methods) yields:

2 $$\frac{\partial {{{{{\rm{\delta }}}}}}{{{{{{\bf{Z}}}}}}}_{\perp }^{-}}{\partial t}+\left({{{{{{\bf{V}}}}}}}_{{{{{{\rm{A}}}}}}0}\cdot {{{{{\boldsymbol{\nabla }}}}}}\right){{{{{\rm{\delta }}}}}}{{{{{{\bf{Z}}}}}}}_{\perp }^{-}=-\left({{{{{{\rm{\delta }}}}}}{{{{{\bf{Z}}}}}}}^{+}\cdot {{{{{\boldsymbol{\nabla }}}}}}\right){{{{{\rm{\delta }}}}}}{{{{{{\bf{Z}}}}}}}_{\perp }^{-}+{T}_{{{{{{\rm{comp}}}}}}}.$$

**Fig. 5:**
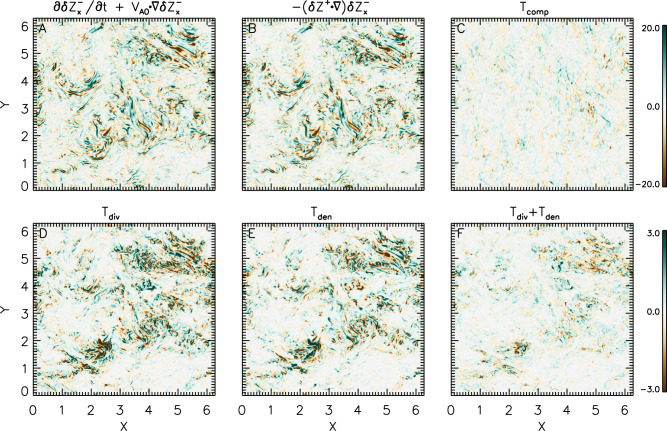
Distributions of the terms in the compressive governing equation (Eq. (2)) of the perpendicular fluctuating Elsässer variable ${\rm {\delta}} {\rm {{Z}}}_{x}^{-}$ at time = 4.0. **A** The evolution term plus the linear term, **B** the nonlinear term, **C** the generalized compressible term ${T}_{{{{{{\rm{comp}}}}}}}$ (Eq. (1)), **D** the term ${T}_{{{{{{\rm{div}}}}}}}$ (Eq. (3)), **E** the term ${T}_{{{{{{\rm{den}}}}}}}$ (Eq. (4)), **F** ${T}_{{{{{{\rm{div}}}}}}}+{T}_{{{{{{\rm{den}}}}}}}$. The displayed variables are dimensionless.

In this equation, the parallel components of ${{{{{\rm{\delta }}}}}}{{{{{{\bf{Z}}}}}}}_{\perp }^{\pm }$ are taken into consideration. The effects of compressibility are embodied in the generalized compressible term ${T}_{{{{{{\rm{comp}}}}}}}$, which acts as a source for ${{{{{\rm{\delta }}}}}}{{{{{{\bf{Z}}}}}}}_{\perp }^{-}$. To understand the generation of ${{{{{\rm{\delta }}}}}}{{{{{{\bf{Z}}}}}}}_{\perp }^{-}$, the top panels of Fig. 5 show the distributions of the left-hand side of Eq. (2), the nonlinear term, and ${T}_{{{{{{\rm{comp}}}}}}}$ for one component of the perpendicular fluctuating Elsässer variable, ${{{{{\rm{\delta }}}}}}{{{{{{\rm{Z}}}}}}}_{x}^{-}$. The variation of $\partial {{{{{\rm{\delta }}}}}}{{{{{{\rm{Z}}}}}}}_{x}^{-}/\partial t+\left({{{{{{\bf{V}}}}}}}_{{{{{{\rm{A}}}}}}0}\cdot {{{{{\boldsymbol{\nabla }}}}}}\right){{{{{\rm{\delta }}}}}}{{{{{{\rm{Z}}}}}}}_{x}^{-}$ is co-located with the variation of the nonlinear term $-\left({{{{{{\rm{\delta }}}}}}{{{{{\bf{Z}}}}}}}^{+}\cdot {{{{{\boldsymbol{\nabla }}}}}}\right){{{{{\rm{\delta }}}}}}{{{{{{\rm{Z}}}}}}}_{x}^{-}$, not with that of the generalized compressible term ${T}_{{{{{{\rm{comp}}}}}}}$.

Figure 5D and E show the terms due to compressibility

3 $${T}_{{{{{{\rm{div}}}}}}}=\frac{{{{{{\rm{\delta }}}}}}{{{{{{\rm{Z}}}}}}}_{x}^{+}-{{{{{\rm{\delta }}}}}}{{{{{{\rm{Z}}}}}}}_{x}^{-}}{8}{{{{{\boldsymbol{\nabla }}}}}}\cdot \left(3{{{{{{\rm{\delta }}}}}}{{{{{\bf{Z}}}}}}}^{-}-{{{{{{\rm{\delta }}}}}}{{{{{\bf{Z}}}}}}}^{+}\right),$$

and due to the density fluctuations

4 $${T}_{{{{{{\rm{den}}}}}}}=-\frac{{{{{{\rm{\delta }}}}}}{{{{{{\rm{Z}}}}}}}_{x}^{+}-{{{{{\rm{\delta }}}}}}{{{{{{\rm{Z}}}}}}}_{x}^{-}}{2}{{{{{\boldsymbol{\nabla }}}}}}\cdot {{{{{{\bf{V}}}}}}}_{{{{{{\rm{A}}}}}}0}.$$

The values of ${T}_{{{{{{\rm{div}}}}}}}$ and ${T}_{{{{{{\rm{den}}}}}}}$ are far smaller than those of the nonlinear term. In many places, ${T}_{{{{{{\rm{div}}}}}}}$ and ${T}_{{{{{{\rm{den}}}}}}}$ are anti-correlated and cancel each other out, which can be readily seen from the distribution of their sum ${T}_{{{{{{\rm{div}}}}}}}+{T}_{{{{{{\rm{den}}}}}}}$ (Fig. 5F). Synthesizing subfigures of Fig. 5 demonstrates that the nonlinear interactions between ${{{{{{\rm{\delta }}}}}}{{{{{\bf{Z}}}}}}}^{-}$ and ${{{{{{\rm{\delta }}}}}}{{{{{\bf{Z}}}}}}}^{+}$ dominate over the compressibility-induced reflection of ${{{{{{\rm{\delta }}}}}}{{{{{\bf{Z}}}}}}}^{+}$ in creating ${{{{{{\rm{\delta }}}}}}{{{{{\bf{Z}}}}}}}^{-}$.

**Fig. 6:**
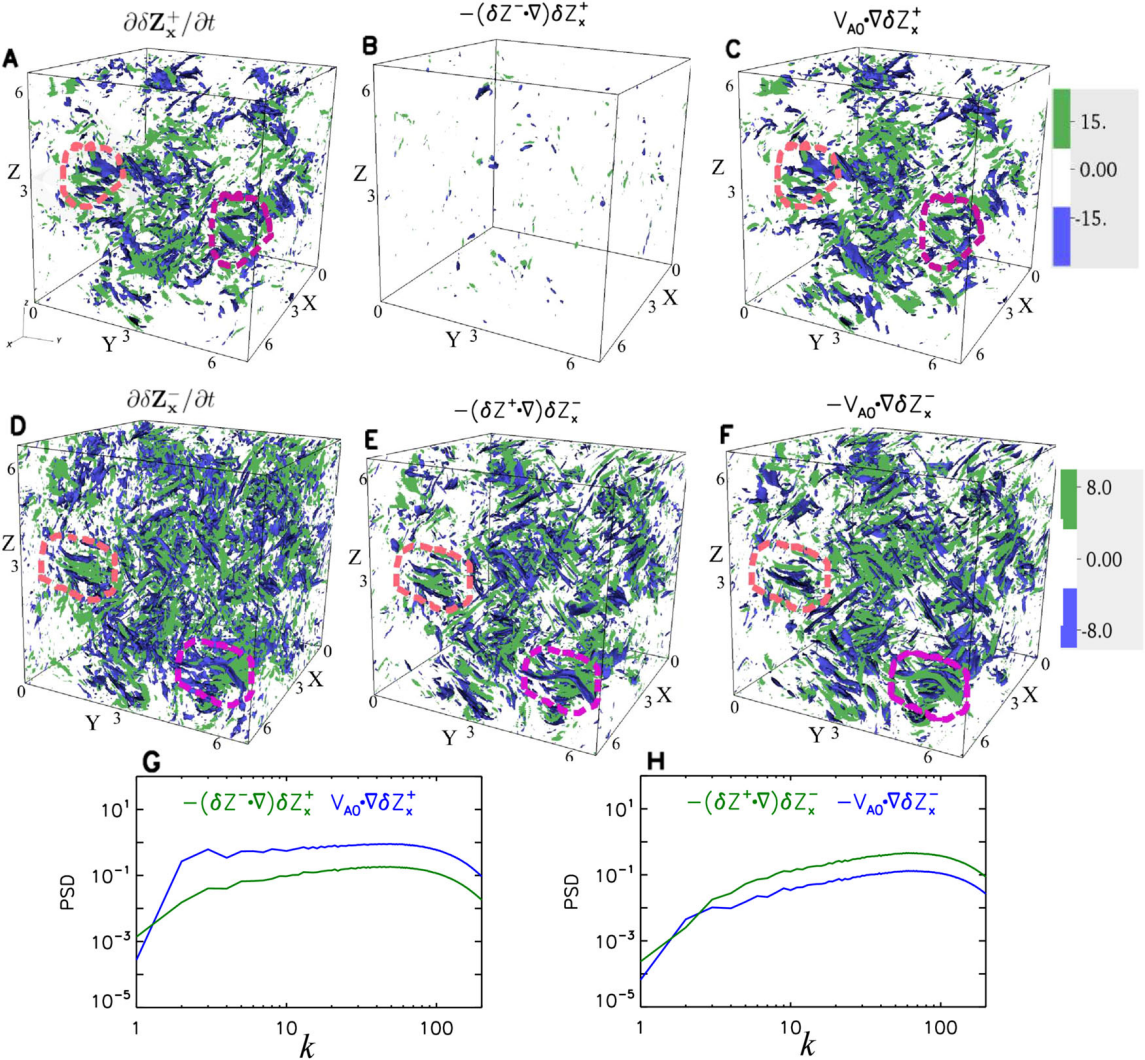
Contributions of nonlinear and linear terms to the variations of the fluctuating Elsässer variables at time = 4.0. **A**–**C** The distributions of the evolution term (**A**), the nonlinear term (**B**), and the linear term (**C**) in the simulation domain for ${{{{{\rm{\delta }}}}}}{{{{{{\rm{Z}}}}}}}_{x}^{+}$. **D**–**F** The distributions of the evolution term (**D**), the nonlinear term (**E**), and the linear term (**F**) in the simulation domain for ${{{{{{\rm{\delta }}}}}}{{{{{\rm{Z}}}}}}}_{x}^{-}$. The orange-dashed and pink-dashed circles highlight the regions with good correlations between the terms. **G** PSDs of $-\left({{{{{{\rm{\delta }}}}}}{{{{{\bf{Z}}}}}}}^{-}\cdot {{{{{\boldsymbol{\nabla }}}}}}\right){{{{{\rm{\delta }}}}}}{{{{{{\rm{Z}}}}}}}_{x}^{+}$ (green line) and $\left({{{{{{\bf{V}}}}}}}_{{{{{{\bf{A}}}}}}{{{{{\bf{0}}}}}}}\cdot {{{{{\boldsymbol{\nabla }}}}}}\right){{{{{\rm{\delta }}}}}}{{{{{{\rm{Z}}}}}}}_{x}^{+}$ (blue line) as a function of wavenumber $k$. **H** PSDs of $-\left({{{{{{\rm{\delta }}}}}}{{{{{\bf{Z}}}}}}}^{+}\cdot {{{{{\boldsymbol{\nabla }}}}}}\right){{{{{\rm{\delta }}}}}}{{{{{{\rm{Z}}}}}}}_{x}^{-}$ (green line) and $\left({{{{{{\bf{V}}}}}}}_{{{{{{\bf{A}}}}}}{{{{{\bf{0}}}}}}}\cdot {{{{{\boldsymbol{\nabla }}}}}}\right){{{{{\rm{\delta }}}}}}{{{{{{\rm{Z}}}}}}}_{x}^{-}$ (blue line) as a function of wavenumber $k$. Their temporal propagation and evolution for the time from 4.0 until 6.0 are shown in Supplementary Movie 2. The displayed variables are dimensionless.

The left panels of Fig. 6, Supplementary Fig. 6 and Supplementary Movie 2 clearly show that the local time derivative $\partial {{{{{\rm{\delta }}}}}}{{{{{{\rm{Z}}}}}}}_{x}^{+}/\partial t$ correlates with and is mainly determined by the linear term $\left({{{{{{\bf{V}}}}}}}_{{{{{{\bf{A}}}}}}{{{{{\bf{0}}}}}}}\cdot {{{{{\boldsymbol{\nabla }}}}}}\right){{{{{\rm{\delta }}}}}}{{{{{{\rm{Z}}}}}}}_{x}^{+}$ at times between 4.0 and 6.0. The linear term is much greater than the nonlinear term $-\left({{{{{{\rm{\delta }}}}}}{{{{{\bf{Z}}}}}}}^{{{{{{\boldsymbol{-}}}}}}}\cdot {{{{{\boldsymbol{\nabla }}}}}}\right){{{{{\rm{\delta }}}}}}{{{{{{\rm{Z}}}}}}}_{x}^{+}$ in amplitude at wavenumbers *k* > 2. These features suggest that ${{{{{\rm{\delta }}}}}}{{{{{{\rm{Z}}}}}}}_{x}^{+}$ behaves as anti-parallel propagating Alfvén waves while retaining a power-law spectral profile.

For ${{{{{\rm{\delta }}}}}}{{{{{{\rm{Z}}}}}}}_{x}^{-}$ shown in the right panels of Fig. 6 and Supplementary Movie 2, we observe a clear positive correlation between $\partial {{{{{\rm{\delta }}}}}}{{{{{{\rm{Z}}}}}}}_{x}^{-}/\partial t$ and $-\left({{{{{{\rm{\delta }}}}}}{{{{{\bf{Z}}}}}}}^{{{{{{\boldsymbol{+}}}}}}}\cdot {{{{{\boldsymbol{\nabla }}}}}}\right){{{{{\rm{\delta }}}}}}{{{{{{\rm{Z}}}}}}}_{x}^{-}$ (compare Fig. 6B and D) as well as between $\left({{{{{{\bf{V}}}}}}}_{{{{{{\bf{A}}}}}}{{{{{\bf{0}}}}}}}\cdot {{{{{\boldsymbol{\nabla }}}}}}\right){{{{{\rm{\delta }}}}}}{{{{{{\rm{Z}}}}}}}_{x}^{-}$ and $-\left({{{{{{\rm{\delta }}}}}}{{{{{\bf{Z}}}}}}}^{{{{{{\boldsymbol{+}}}}}}}\cdot {{{{{\boldsymbol{\nabla }}}}}}\right){{{{{\rm{\delta }}}}}}{{{{{{\rm{Z}}}}}}}_{x}^{-}$ (compare Fig. 6F and D). The magnitude of the nonlinear term is the largest and about twice the amplitude of the other terms. This suggests that the nonlinear term acts as the source term for the generation of the variations of the other two terms and make ${{{{{{\rm{\delta }}}}}}{{{{{\bf{Z}}}}}}}^{-}$ “sail along” ${{{{{{\rm{\delta }}}}}}{{{{{\bf{Z}}}}}}}^{+}$ at speed ${-V}_{{{{{{\rm{A}}}}}}0}$. The prevailing anti-correlation between $\partial {{{{{\rm{\delta }}}}}}{{{{{{\rm{Z}}}}}}}_{x}^{-}/\partial {{{{{\rm{t}}}}}}$ and $-\left({{{{{{\bf{V}}}}}}}_{{{{{{\bf{A}}}}}}{{{{{\bf{0}}}}}}}\cdot {{{{{\boldsymbol{\nabla }}}}}}\right){{{{{\rm{\delta }}}}}}{{{{{{\rm{Z}}}}}}}_{x}^{-}$ (compare Fig. 6B and F) reveals again that ${{{{{{\rm{\delta }}}}}}{{{{{\bf{Z}}}}}}}^{{{{{{\boldsymbol{-}}}}}}}$ no longer represents classical, parallel-propagating Alfvén waves. ${{{{{{\rm{\delta }}}}}}{{{{{\bf{Z}}}}}}}^{-}$ appears to “sail along” ${{{{{{\rm{\delta }}}}}}{{{{{\bf{Z}}}}}}}^{+}$ as a result of the nonlinear term continuously generating ${{{{{{\rm{\delta }}}}}}{{{{{\bf{Z}}}}}}}^{-}$, and ${{{{{{\rm{\delta }}}}}}{{{{{\bf{Z}}}}}}}^{-}$ does not represent a kind of waves but a kind of anomalous fluctuations propagating in the same direction as ${{{{{{\rm{\delta }}}}}}{{{{{\bf{Z}}}}}}}^{+}$.

The co-traveling ${{{{{{\rm{\delta }}}}}}{{{{{\bf{Z}}}}}}}^{-}$ is continuously generated through the nonlinearity in our work. It is seemingly similar but essentially different from the anomalous ${{{{{{\rm{\delta }}}}}}{{{{{\bf{Z}}}}}}}^{-}$ produced through the linear reflection of Alfvén waves at background inhomogeneities discussed in previous work^44–49^. The apparent similarity between the two is that the (apparent) propagating direction of the two is the same as that of ${{{{{{\rm{\delta }}}}}}{{{{{\bf{Z}}}}}}}^{+}$, and both can be produced continuously under appropriate conditions. The essential differences between them are: (1) The origin mechanism is different. The anomalous ${{{{{{\rm{\delta }}}}}}{{{{{\bf{Z}}}}}}}^{-}$ in our work is driven by nonlinear coupling with ${{{{{{\rm{\delta }}}}}}{{{{{\bf{Z}}}}}}}^{+}$; (2) The evolution process is different. In previous work, since ${{{{{{\rm{\delta }}}}}}{{{{{\bf{Z}}}}}}}^{-}$ is generated by reflection, it leaves the driving source and propagates in the opposite direction after generation, which is in analogy to smoke coming out of a ship’s smokestack^46^. In this work, ${{{{{{\rm{\delta }}}}}}{{{{{\bf{Z}}}}}}}^{-}$ continues to travel with the driving ${{{{{{\rm{\delta }}}}}}{{{{{\bf{Z}}}}}}}^{+}$ and further facilitates the nonlinear cascade of its parent ${{{{{{\rm{\delta }}}}}}{{{{{\bf{Z}}}}}}}^{+}$ and itself. In order to distinguish these two processes, we refer to the anomalous ${{{{{{\rm{\delta }}}}}}{{{{{\bf{Z}}}}}}}^{-}$ component in our work as the NCI-anomalous ${{{{{{\rm{\delta }}}}}}{{{{{\bf{Z}}}}}}}^{-}$ (where NCI stands for “nonlinear-coupling-induced”). We refer to the reflection-driven anomalous ${{{{{{\rm{\delta }}}}}}{{{{{\bf{Z}}}}}}}^{-}$ in former work as the LRI-anomalous ${{{{{{\rm{\delta }}}}}}{{{{{\bf{Z}}}}}}}^{-}$ (where LRI stands for “linear-reflection-induced”).

For the current simulation, the plasma $\beta (={P}_{{{{{{\rm{th}}}}}}}/{P}_{{{{{{\rm{mag}}}}}}})$ is about 1.4. To understand the influence of $\beta$ or compressible perturbations on the results, we also conduct low $\beta (\approx 0.71)$ and high $\beta (\approx 7.6)$ simulations, in which the relative density fluctuation amplitudes are about 36% and 10%, respectively. In both simulations, the co-propagation of ${{{{{{\rm{\delta }}}}}}{{{{{\bf{Z}}}}}}}^{-}$ with ${{{{{{\rm{\delta }}}}}}{{{{{\bf{Z}}}}}}}^{+}$ generated by the nonlinearities persists, which indicates that $\beta$ and compressive fluctuations have only little influence on our results.

Results of an additional simulation with small initial perturbation (${{{{{{\rm{\delta }}}}}}{B}}_{{{{{{\rm{rms}}}}}}}/{B}_{0}\, \approx \, 0.1$) in Supplementary Fig. 7 reveal that, for ${{{{{{\rm{\delta }}}}}}{{{{{\bf{Z}}}}}}}^{+}$, the linear term still dominates to make ${{{{{{\rm{\delta }}}}}}{{{{{\bf{Z}}}}}}}^{+}$ propagate at ${-{V}}_{{{{{{\rm{A}}}}}}0}$. For ${{{{{{\rm{\delta }}}}}}{{{{{\bf{Z}}}}}}}^{-}$, whose propagation direction is now opposite to that of ${{{{{{\rm{\delta }}}}}}{{{{{\bf{Z}}}}}}}^{+}$, the nonlinear term no longer determines ${{{{{{\rm{\delta }}}}}}{{{{{\bf{Z}}}}}}}^{-}$, and in many regions of the simulation domain, compressibility effects are responsible for the generation of ${{{{{{\rm{\delta }}}}}}{{{{{\bf{Z}}}}}}}^{-}$.

To verify the finding that ${{{{{{\rm{\delta }}}}}}{{{{{\bf{Z}}}}}}}^{-}$ is mainly produced by the nonlinearity rather than by compressibility, we conduct a decaying imbalanced reduced MHD (RMHD) simulation, which numerically solves Elsässer-potential equations with a fourth-order hyperviscosity dissipation term^10^. The dotted lines in Fig. 2D show the power spectra of ${{{{{\rm{\delta }}}}}}{{{{{{\bf{Z}}}}}}}^{\pm }$ at ${{{{{\rm{time}}}}}}=4.0$ from our imbalanced RMHD simulation, which closely resemble those in the compressive MHD case (the solid lines in Fig. 2D). Nevertheless, the power of ${{{{{{\rm{\delta }}}}}}{{{{{\bf{Z}}}}}}}^{-}$ in the compressive MHD simulation is slightly higher than that in RMHD simulation. We attribute this discrepancy to the initial seeding of the minority Elsässer variable by compressibility at early times of the simulation (Supplementary Fig. 13).

In previous simulations of imbalanced Alfvénic turbulence in the RMHD regime, Perez and Boldyrev^50^ find that Elsässer energy spectra have different amplitudes but the same scaling with an index of −3/2, while Beresnyak and Lazarian^23^ show that the spectrum of the majority component is steeper than that of the minority component (spectral slopes between −1.12 and −1.93). The spectral slopes obtained from our simulations of imbalanced Alfvénic turbulence are similar to those observed in specific intervals of PSP measurements.

In our imbalanced RMHD simulation, ${{{{{{\rm{\delta }}}}}}{{{{{\bf{Z}}}}}}}^{+}$ and ${{{{{{\rm{\delta }}}}}}{{{{{\bf{Z}}}}}}}^{-}$ propagate in the same direction (see Supplementary Fig. 9). For ${{{{{{\rm{\delta }}}}}}{{{{{\bf{Z}}}}}}}^{+}$, the linear term dominates. For ${{{{{{\rm{\delta }}}}}}{{{{{\bf{Z}}}}}}}^{-}$, $\partial {{{{{\rm{\delta }}}}}}{{{{{{\rm{Z}}}}}}}_{x}^{-}/\partial t$ correlates closely with $-\left({{{{{{\boldsymbol{\delta }}}}}}{{{{{\bf{Z}}}}}}}^{+}\cdot {{{{{\boldsymbol{\nabla }}}}}}\right){{{{{\rm{\delta }}}}}}{{{{{{\rm{Z}}}}}}}_{x}^{-}$, but has an inverse relationship with $-\left({{{{{{\bf{V}}}}}}}_{{{{{{\bf{A}}}}}}{{{{{\bf{0}}}}}}}\cdot {{{{{\boldsymbol{\nabla }}}}}}\right){{{{{\rm{\delta }}}}}}{{{{{{\rm{Z}}}}}}}_{x}^{-}$, which reveals that in imbalanced RMHD turbulence, the nonlinear term also acts as the source term to generate ${{{{{{\rm{\delta }}}}}}{{{{{\rm{Z}}}}}}}^{-}$ that co-propagates with δ**Z**^+^.

### Scale locality of the cascade

**Fig. 7:**
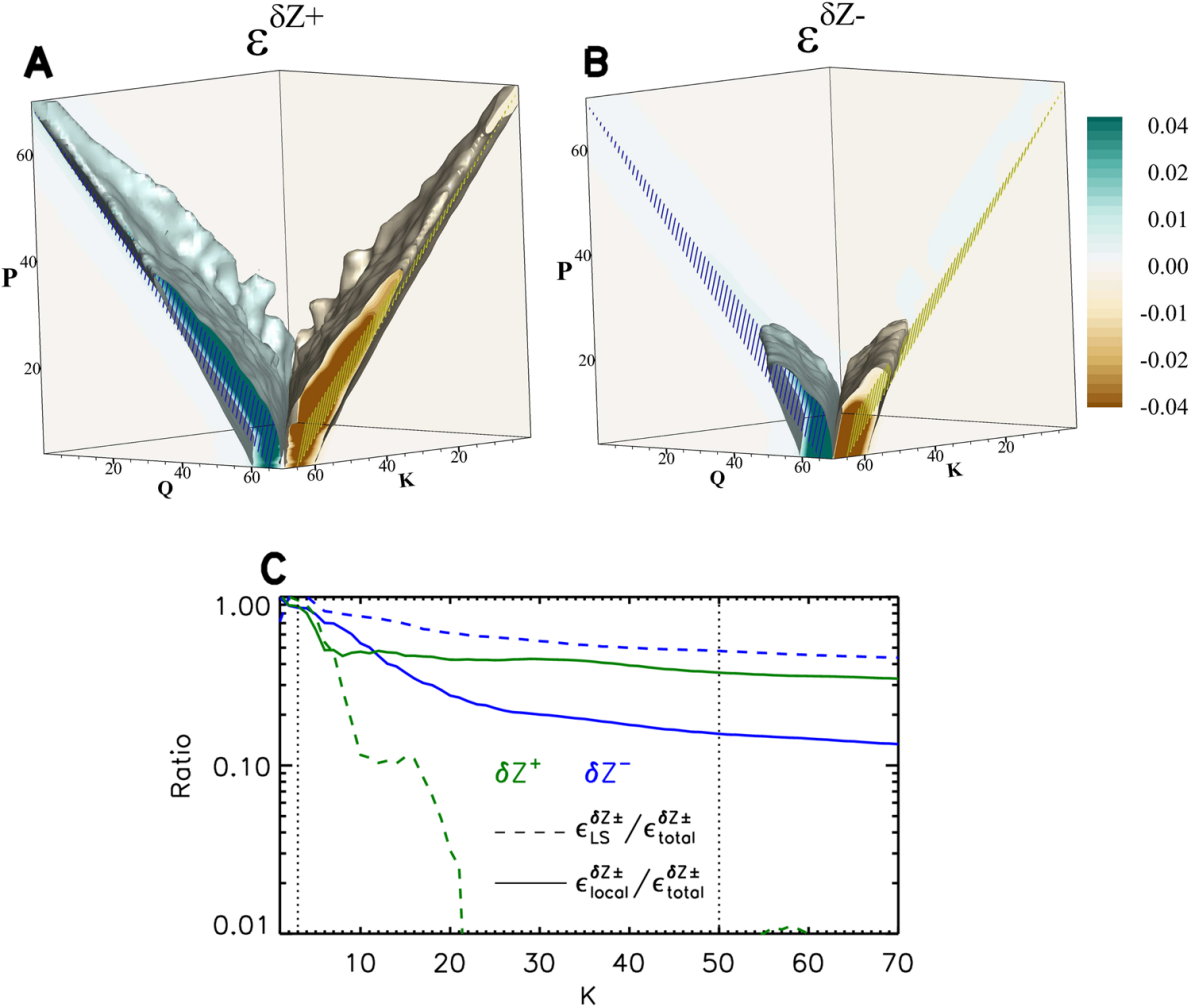
Rates of energy transfer between different scales at time = 4.0. **A** and **B** Distributions of energy transfer rates, ${\varepsilon }^{{{\delta }{Z}}^{{{{{{\boldsymbol{\pm }}}}}}}},$ from shell $Q$ to shell $K$ by mediation of $P$ modes in $K-Q-P$ space (**A** for ${{{{{{\rm{\delta }}}}}}{{{{{\bf{Z}}}}}}}^{{{{{{\boldsymbol{+}}}}}}}$ and **B** for ${{{{{{\rm{\delta }}}}}}{{{{{\bf{Z}}}}}}}^{{{{{{\boldsymbol{-}}}}}}}$). The light blue and light brown surfaces denote isosurfaces of ${\varepsilon }^{{{\delta }{Z}}^{{{{{{\boldsymbol{\pm }}}}}}}}$ with the values of −0.01 and 0.01, respectively. The planes filled with blue and yellow stripes denote the relations $K=Q+P$, and $Q=K+P$, respectively. **C** Ratios of the energy transfer rate by large-scale interactions (dashed lines) and by local interactions (solid lines) to the transfer rate due to all triadic interactions on a band of $K$-shells for ${{{{{{\rm{\delta }}}}}}{{{{{\bf{Z}}}}}}}^{{{{{{\boldsymbol{+}}}}}}}$ (green lines) and ${{{{{{\rm{\delta }}}}}}{{{{{\bf{Z}}}}}}}^{{{{{{\boldsymbol{-}}}}}}}$ (blue lines). The two vertical dotted lines indicate the inertial range of the simulated turbulence. The displayed variables are dimensionless.

Lastly, we employ wavevector dynamics analysis to investigate the energy transfer across various scales , aiming to scrutinize the scale locality or non-locality of the nonlinear interactions of ${{{{{{\rm{\delta }}}}}}{{{{{\bf{Z}}}}}}}^{{{{{{\boldsymbol{+}}}}}}}$ and ${{{{{{\rm{\delta }}}}}}{{{{{\bf{Z}}}}}}}^{{{{{{\boldsymbol{-}}}}}}}$. We estimate the energy transfer rate $\varepsilon (K,Q,P)$ of ${{{{{{\rm{\delta }}}}}}{{{{{\bf{Z}}}}}}}^{{{\pm }}}$ from the energy in wavenumber shell $Q$ to the energy in wavenumber shell *K* due to interactions with ${{{{{{\rm{\delta }}}}}}{{{{{\bf{Z}}}}}}}^{{{{{{\boldsymbol{\mp }}}}}}}$ in wavenumber shell $P$ according to triadic interactions. Figure 7A and B show the distributions of ${\varepsilon }^{{{{{{{\rm{\delta }}}}}}{Z}}^{\pm }}$ in $K-Q-P$ space. Shell $K$ receives energy from shell $Q$ with the help of modes in shell *P*, leading to positive transfer rates in the plane of $K=Q+P$. Negative transfer rates relate that shell $K$ contributes energy to shell $Q$ through interaction with modes in shell *P*. This signal clusters near the plane of $Q=K+P$. In this sense, Fig. 7A and B represent quantitative visualizations of the energy transfer rate due to triadic interactions^14,51^.

A new feature in Fig. 7A and B is that the triadic interactions are different between ${{{{{{\rm{\delta }}}}}}{{{{{\bf{Z}}}}}}}^{+}$ and ${{{{{{\rm{\delta }}}}}}{{{{{\bf{Z}}}}}}}^{-}$. For ${{{{{{\rm{\delta }}}}}}{{{{{\bf{Z}}}}}}}^{+}$ in Fig. 7A, ${{{{{{\rm{\delta }}}}}}{{{{{\bf{Z}}}}}}}^{-}$ modes with $P$ varying from small wavenumbers to large wavenumbers participate in the relevant triadic interactions. For ${{{{{{\rm{\delta }}}}}}{{{{{\bf{Z}}}}}}}^{-}$ in Fig. 7B, only ${{{{{{\rm{\delta }}}}}}{{{{{\bf{Z}}}}}}}^{+}$ modes with small $P$ participate in the triadic interactions. We further observe that the total energy transfer rate for ${{{{{{\rm{\delta }}}}}}{{{{{\bf{Z}}}}}}}^{+}$, ${\varepsilon }_{{\rm {{all}}\; P}}^{{{{{{{\rm{\delta }}}}}}{Z}}^{+}},$ is determined by ${{{{{{\rm{\delta }}}}}}{{{{{\bf{Z}}}}}}}^{-}$ modes with $P\ge 6$, but ${\varepsilon }_{{{{{{\rm{all}}}}}}\; {{{{{\rm{P}}}}}}}^{{{\delta }{Z}}^{-}}$ is dominated by the large-scale interactions with ${{{{{{\rm{\delta }}}}}}{{{{{\bf{Z}}}}}}}^{+}$ modes satisfying $P < 6$ (see Supplementary Fig. 10).

Figure 7C compares the relative contributions of the large-scale interactions and the local-scale interactions at different bands of $K$-shells for ${{{{{{\rm{\delta }}}}}}{{{{{\bf{Z}}}}}}}^{{{\pm }}}$. As $K$ increases, the proportion of the large-scale interactions in the total interactions weakens quickly, and becomes much less than that of the local-scale interactions, suggesting that the large-scale interactions contribute less than the local-scale interactions to the energy transfer of ${{{{{{\rm{\delta }}}}}}{{{{{\bf{Z}}}}}}}^{{{{{{\boldsymbol{+}}}}}}}$. The energy transfer in ${{{{{{\rm{\delta }}}}}}{{{{{\bf{Z}}}}}}}^{{{{{{\boldsymbol{-}}}}}}}$ exhibits the opposite behavior, where the proportion of the large-scale interactions in the total interactions remains greater than that of the local-scale interactions. This finding supports our interpretation that large-scale interactions play a dominant role in the energy cascade of ${{{{{{\rm{\delta }}}}}}{{{{{\bf{Z}}}}}}}^{{{{{{\boldsymbol{-}}}}}}}$. However, for balanced turbulence, the local-scale interactions account for a more substantial fraction of the energy transfer rate than the large-scale interactions (Supplementary Fig. 11). The dominance of the large-scale interactions can be responsible for the ${k}^{-1}$ spectrum of ${{{{{{\rm{\delta }}}}}}{{{{{\bf{Z}}}}}}}^{{{{{{\boldsymbol{-}}}}}}}$ (see Supplementary Discussion). Likewise, the phenomenological treatment of ${{{{{{\rm{\delta }}}}}}{{{{{\bf{Z}}}}}}}^{{{{{{\boldsymbol{+}}}}}}}$ suggests that the local-scale interactions are potentially responsible for the ${k}^{-5/3}$ spectrum of ${{{{{{\rm{\delta }}}}}}{{{{{\bf{Z}}}}}}}^{{{{{{\boldsymbol{+}}}}}}}$ (see Supplementary Discussion).

**Fig. 8:**
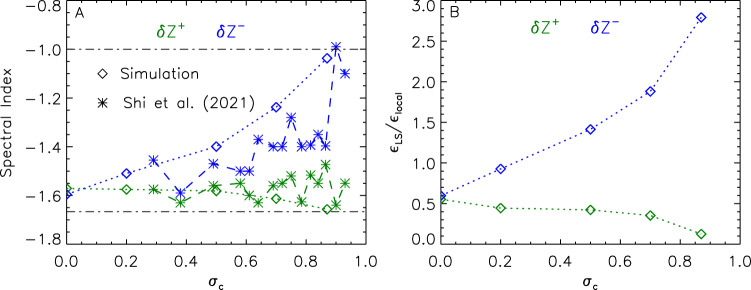
Effects of the normalized cross helicity. **A** on the spectral index of ${{{{{\rm{\delta }}}}}}{{{{{{\bf{Z}}}}}}}^{{{{{{\boldsymbol{\pm }}}}}}}$ and **B o**n the ratio between the energy transfer due to large-scale interactions (${\varepsilon }_{{{{{{\rm{LS}}}}}}}$) and the energy transfer due to local-scale interactions (${\varepsilon }_{{{{{{\rm{local}}}}}}}$) of ${{{{{\rm{\delta }}}}}}{{{{{{\bf{Z}}}}}}}^{{{{{{\boldsymbol{\pm }}}}}}}$. In **A**, the star symbols represent results from Shi et al.^34^, the upper and lower dotted-dashed lines indicate the spectral indices of ${{{{{\rm{\delta }}}}}}{{{{{{\bf{Z}}}}}}}^{\pm }$ being −1.00 and −1.67, respectively.

To understand the influence of the normalized cross helicity ${\sigma }_{\rm {{c}}}$, we plot the dependence of ${{{{{\rm{\delta }}}}}}{{{{{{\rm{Z}}}}}}}^{\pm }$’s spectral indexes with ${\sigma }_{\rm {{c}}}$ in Fig. 8a, and the dependencies of ratios between the large-scale energy transfer (${\varepsilon }_{{{{{{\rm{LS}}}}}}}$) and the local-scale energy transfer (${\varepsilon }_{{{{{{\rm{local}}}}}}}$) with ${\sigma }_{\rm {{c}}}$ in Fig. 8b. As ${\sigma }_{\rm {{c}}}$ increases, the spectral index of ${{{{{\rm{\delta }}}}}}{{\rm {{Z}}}}^{-}$ rises and approaches −1, while the spectral index of ${{{{{\rm{\delta }}}}}}{{{\rm {Z}}}}^{+}$ stays around −1.6. These variation trends are comparable to the observational findings reported by Shi et al. ^34^. With increasing ${\sigma }_{\rm {{c}}}$, the large-scale interactions take increasingly control of the energy transfer of ${{{{{\rm{\delta }}}}}}{{{\rm {Z}}}}^{-}$. The local-scale interactions, however, continue to play the dominant role in the energy transfer of ${{{{{\rm{\delta }}}}}}{{{\rm {Z}}}}^{+}$.

## Discussion

Our work suggests that the energy transfer in imbalanced Alfvénic turbulence is completed by both local and large-scale interactions between Alfvén waves (${{{{{{\rm{\delta }}}}}}{{{{{\bf{Z}}}}}}}^{+}$) and anomalous fluctuations (${{{{{{\rm{\delta }}}}}}{{{{{\bf{Z}}}}}}}^{-}$) that propagate in the same direction as the waves. Although a critical balance^3,52,53^ between linear and nonlinear timescales is predicted to exist for balanced turbulence (Supplementary Fig. 8), we do not find a critical balance between the linear and nonlinear terms in imbalanced Alfvénic turbulence. Instead, linearity dominates over nonlinearity for ${{{{{{\rm{\delta }}}}}}{{{{{\bf{Z}}}}}}}^{+}$ while nonlinearity dominates over linearity for ${{{{{{\rm{\delta }}}}}}{{{{{\bf{Z}}}}}}}^{-}$.

During PSP*’s* first encounter, the relative density variation is typically in the range of 0.1–0.2, which suggests that the slow solar wind probed by the PSP is not completely incompressible^47^. In this work, we use the fully compressible MHD framework to reproduce the solar wind fluctuations with high Alfvénicity and low to intermediate density fluctuations. Our simulations start from an incompressible, highly Alfvénic state and finally converge to a weakly compressive regime. Through the use of incompressible initial conditions, we prime our simulations to converge towards incompressible solutions as well^54–56^. Numerical simulations by Matthaeus et al. ^57^ show that the level of compressive fluctuations depends critically on the initialization. Moreover, high Alfvénic correlations tend to suppress the generation of compressive modes^39,58,59^.

During the initial stage of our compressible MHD simulation, the ponderomotive force, associated with the spatial variation of the total magnetic field magnitude, triggers density perturbations. Consequently, compressibility plays a crucial role in shaping the precursor dynamics in our analysis. As illustrated in Supplementary Fig. 13, at ${{{{{\rm{time}}}}}}=1.0$, the compressibility induces density variations in some regions. The divergence of the velocity field exhibits a comparable magnitude to the curl of the velocity field in numerous regions of the simulation domain. Similarly, the compressible terms contribute to the generation of minority Elsässer variables at some locations. Hence, compressibility plays an important role in establishing the conditions during the initial stage. However, as the system evolves, the gradient of the magnetic pressure gradually assumes balance with the thermal pressure gradient, limiting the ability of the ponderomotive force to induce additional compressible effects. Consequently, the influence of compressibility diminishes gradually and, more significantly, during the late-stage evolution.

When we start the compressible simulation with unidirectionally-propagating Alfvén waves (cross-helicity of 1 or −1), the ponderomotive force is the only way to generate ${{{{{{\rm{\delta }}}}}}{{{{{\bf{Z}}}}}}}^{-}$. As ${{{{{{\rm{\delta }}}}}}{{{{{\bf{Z}}}}}}}^{-}$ becomes large enough, the nonlinearity comes into play to seed ${{{{{{\rm{\delta }}}}}}{{{{{\bf{Z}}}}}}}^{-}$. Meanwhile, the influence of the ponderomotive force weakens as multiscale pressure-balanced structures form. As a result, the nonlinearity continues to act as a source term to produce ${{{{{{\rm{\delta }}}}}}{{{{{\bf{Z}}}}}}}^{-}$ that travels along with ${{{{{{\rm{\delta }}}}}}{{{{{\bf{Z}}}}}}}^{+}$ in the later dynamics with stabilized power-law profiles in the energy spectra.

The compressible governing equations of the perpendicular Elsässer fields (Supplementary Eqs. (5) and (6)) show that, in the compressive regime, the perpendicular Elsässer variables cannot be considered a mere representation of counter-propagating fluctuations. In this work about imbalanced Alfvénic turbulence with weak compressibility and low-amplitude density fluctuations, both ${{{{{\rm{\delta }}}}}}{{{{{{\bf{Z}}}}}}}_{\perp }^{+}$ and ${{{{{\rm{\delta }}}}}}{{{{{{\bf{Z}}}}}}}_{\perp }^{-}$ propagate with the negative Alfvén velocity. Considering the fact that nonlinear interactions with large-scale ${{{{{\rm{\delta }}}}}}{{{{{{\bf{Z}}}}}}}^{+}$ fluctuations can be stronger than those with local-scale ${{{{{\rm{\delta }}}}}}{{{{{{\bf{Z}}}}}}}^{+}$ fluctuations, the non-local coupling with large-scale ${{{{{\rm{\delta }}}}}}{{{{{{\bf{Z}}}}}}}^{+}$ fluctuations favors the change of the propagation direction of ${{{{{\rm{\delta }}}}}}{{{{{{\bf{Z}}}}}}}^{-}$.

In our study of homogeneous imbalanced MHD turbulence, we find a source of ${{{{{{\rm{\delta }}}}}}{{{{{\bf{Z}}}}}}}^{-}$, which is the nonlinearity and co-exists with other sources of ${{{{{{\rm{\delta }}}}}}{{{{{\bf{Z}}}}}}}^{-}$, like those known from inhomogeneous reflection-driven MHD turbulence. This source is local and persists, and thus is complementary to the source due to reflection, which depends on the large-scale inhomogeneity of the background. We justify that NCI-anomalous ${{{{{{\rm{\delta }}}}}}{{{{{\bf{Z}}}}}}}^{-}$ propagates along with ${{{{{{\rm{\delta }}}}}}{{{{{\bf{Z}}}}}}}^{+}$, and the coherent interaction between co-traveling ${{{{{{\rm{\delta }}}}}}{{{{{\bf{Z}}}}}}}^{\pm }$ sustains imbalanced weak compressible MHD turbulence as well as imbalanced reduced MHD turbulence, which are often considered a result of the collisions between counter-propagating Alfvén waves.

Fully compressible simulations of imbalanced MHD turbulence and their potential application to solar wind turbulence have received major attention in the literature recently^40,60–64^. The nature of the minority component and its role in the cascade of turbulence are regarded as some of the core questions related to this issue. To understand the turbulent cascade in the solar wind, Grappin et al.^40^ employ simulations of decaying compressible turbulence in three dimensions, considering both homogeneous and expanding cases. In the homogeneous simulations, they observe a steep dominant spectrum and a flat subdominant spectrum, and the final spectral indices depend on the initial cross helicity.

In recent studies by Magyar et al.^63,64^, the coupling and co-propagation of Elsässer variables are proposed as a new phenomenon in MHD simulations, yet the setup in their model is a special case, which requires waves to propagate on transverse inhomogeneities as surface Alfvén waves or kink waves. In our study, the transverse inhomogeneity of the Alfvén speed is generated due to nonlinear processes. However, this inhomogeneity is weak and thus inefficient in the development of surface waves as those proposed by Magyar et al. ^63^. For surface waves, the total pressure perturbations act as sources for ${{{{{\rm{\delta }}}}}}{{{{{{\bf{Z}}}}}}}_{\perp }^{\pm }$, and thus couple them to propagate at the same speed. In our case, owing to the formation of multiscale pressure-balanced structures, the total pressure perturbations are small and unlikely to play a crucial role in creating ${{{{{\rm{\delta }}}}}}{{{{{{\bf{Z}}}}}}}_{\perp }^{\pm }$ (Supplementary Fig. 2, Fig. 5 and Supplementary Fig. 6). For surface waves, ${{{{{\rm{\delta }}}}}}{{{{{{\bf{Z}}}}}}}_{\perp }^{\pm }$ do not travel at ${{{{{{\rm{|}}}}}}{{{{{\bf{V}}}}}}}_{{{{{{\rm{A}}}}}}0}{{{{{\rm{|}}}}}}$, while the propagation speed of ${{{{{\rm{\delta }}}}}}{{{{{{\bf{Z}}}}}}}_{\perp }^{\pm }$ is ${{{{{{\boldsymbol{-}}}}}}{{{{{\bf{|V}}}}}}}_{{{{{{\rm{A}}}}}}0}{{{{{\rm{|}}}}}}$ in our case (Fig. 4).

For $\beta \le 1$, the model of nearly incompressible MHD turbulence comprises a superposition of a majority population of zero-frequency 2D fluctuations plus the minority population of slab fluctuations. The non-compressible slab fluctuations correspond to a combination of counter-propagating Alfvén modes^65^, and the Elsässer variables ${{{{{\rm{\delta }}}}}}{{{{{{\bf{Z}}}}}}}_{\perp }^{\pm }$ contain both 2D and slab components^56^. In our model, weak compressible imbalanced MHD turbulence also has two components, but the dominant component consists of unidirectionally-propagating Alfvén waves (${{{{{\rm{\delta }}}}}}{{{{{{\bf{Z}}}}}}}_{\perp }^{+}$) and the sub-dominant component is the NCI-anomalous ${{{{{\rm{\delta }}}}}}{{{{{{\bf{Z}}}}}}}_{\perp }^{-}$, which co-propagates with ${{{{{\rm{\delta }}}}}}{{{{{{\bf{Z}}}}}}}_{\perp }^{+}$.

To overcome the shortcoming of 1D observed time series of solar wind fluctuations, we use 4D spatial–temporal simulation data to identify the propagation directions. In order to clearly diagnose the propagation characteristics of Elsässer variables observationally, we require multiple satellites to form a constellation with inter-spacecraft distances on MHD scales. At present, constellation missions are not able to meet the above measurement requirement since, for example,  the Cluster-II spacecraft are typically spaced near the ion kinetic scales  and the Magnetospheric Multiscale (MMS) spacecraft are typically spaced near electron kinetic scales^66,67^. In the future, to really solve the mystery of the Elsässer variables in solar wind turbulence, we call for considering a satellite constellation program with spacing distances including MHD scales. Future constellation mission concepts with many spacecraft (e.g., HelioSwarm and AME)^68,69^, if implemented, are expected to finally settle the critical issue of Alfvénic turbulence in the heliosphere.

We analyze the case of imbalanced Alfvénic turbulence with high cross helicity. The spectral slopes for ${{{{{\rm{\delta }}}}}}{{{{{{\bf{Z}}}}}}}^{+}$ and ${{{{{\rm{\delta }}}}}}{{{{{{\bf{Z}}}}}}}^{-}$ change with cross helicity. An important prospect for future studies is the variation of the nonlinear interactions with Alfvénicity. Another issue is that we use the background magnetic field as the propagation direction since we apply statistical methods to study the collective behavior of the turbulence. A local magnetic field averaged over a local neighborhood is potentially better suited as the propagation direction when determining anisotropy. It would also be interesting to study the role of large-scale interactions versus a passive cascade in shaping the power-law spectrum of compressive fluctuations, which might be related to slow-mode-like compressive fluctuations^70^.

We have taken a step forward in understanding the essence of imbalanced Alfvénic turbulence. A broader topic of interest is the open question as to how imbalanced Alfvénic turbulence evolves along with the solar wind’s expansion into the heliosphere. It may experience complex non-radial expansion and stream interactions^34^, but these lie beyond the scope of this work. The initial condition of ${{{{{{\rm{\delta }}}}}}{{{{{\bf{Z}}}}}}}^{-}$ set in this paper may be produced through other mechanisms during the evolution of solar wind turbulence. Combined with the observations by PSP and Solar Orbiter, our mechanism of energy transfer has the potential to be  an important step to settle the puzzle of solar wind heating and acceleration as well as heliosphere formation within the local interstellar medium.

## Methods

### Observations by the Parker Solar Probe (PSP)

We studied PSP data on 6 November 2018 when PSP was near its first perihelion. The data of magnetic fields were acquired from the fluxgate magnetometer of the FIELDS instrument suite^71^ with a sampling rate of 290 vectors s^−1^. The data of proton number density and fluid velocity were obtained from the Solar Probe Cup of the SWEAP instrument suite at a cadence of about 0.58 s^72^.

To investigate the spectra of the solar wind turbulent fluctuations and examine their stationary randomness, we first divide the one day of magnetic field, proton velocity, and proton density data into 24 one-hour intervals, and then employ a fast Fourier transform to each interval to calculate the respective trace power spectral densities (PSDs). We find that the PSD profiles in the frequency range $0.001\,{{{{{\rm{Hz}}}}}}\le f\le 0.1\,{{{{{\rm{Hz}}}}}}$ can be approximated with a single power law. Therefore, we adopt a linear fit on log-log scales to estimate the power-law indices of each interval.

The spectral indices of the PSDs for the perturbed plasma velocity ${{{{{\rm{\delta }}}}}}{{{{{\bf{V}}}}}}$, the perturbed magnetic field ${{{{{\rm{\delta }}}}}}{{{{{\bf{B}}}}}}$, the fluctuating Elsässer variables ${{{{{\rm{\delta }}}}}}{{{{{{\bf{Z}}}}}}}^{+}\left(\right.={{{{{\rm{\delta }}}}}}{{{{{\bf{V}}}}}}{+}{{{{{\rm{\delta }}}}}}{{{{{{\bf{V}}}}}}}_{{{{{{\rm{A}}}}}}},$ with ${{{{{\rm{\delta }}}}}}{{{{{{\bf{V}}}}}}}_{{{{{{\rm{A}}}}}}}={{{{{\rm{\delta }}}}}}{{{{{\bf{B}}}}}}{/}\sqrt{\rho }$, ${\rho }$ being density) and ${{{{{\rm{\delta }}}}}}{{{{{{\bf{Z}}}}}}}^{-}\left(={{{{{\rm{\delta }}}}}}{{{{{\bf{V}}}}}}{-}{{{{{\rm{\delta }}}}}}{{{{{{\bf{V}}}}}}}_{{{{{{\rm{A}}}}}}}\right)$ stay stable, which indicates the stationary randomness of the different variables.

### Numerical simulation

The compressible MHD equations are written in the following nondimensional form:

5 $$\frac{\partial \rho }{\partial t}+{{{{{\boldsymbol{\nabla }}}}}}\cdot \left(\rho {{{{{\bf{V}}}}}}\right)=0,$$

6 $$\frac{\partial \rho {{{{{\bf{V}}}}}}}{\partial t}+{{{{{\boldsymbol{\nabla }}}}}}\cdot \left(\rho {{{{{\bf{VV}}}}}}+\left({P}_{{{{{{\rm{th}}}}}}}+\frac{1}{2}{B}^{2}\right){{{{{\bf{I}}}}}}-{{{{{\bf{BB}}}}}}\right)=\nu {\nabla }^{2}{{{{{\bf{V}}}}}},$$

7 $$\frac{\partial e}{\partial {{{{{\rm{t}}}}}}}+{{{{{\boldsymbol{\nabla }}}}}}\cdot \left[{{{{{\bf{V}}}}}}\left(e+p+\frac{1}{2}{B}^{2}\right)-\left({{{{{\bf{V}}}}}}\cdot {{{{{\bf{B}}}}}}\right){{{{{\bf{B}}}}}}\right]={{{{{\boldsymbol{\nabla }}}}}}\cdot \left({{{{{\bf{V}}}}}}\cdot \nu \nabla {{{{{\bf{V}}}}}}\right)+{{{{{\boldsymbol{\nabla }}}}}}\cdot \left({{{{{\bf{B}}}}}}\times \eta {{{{{\bf{j}}}}}}\right),$$

8 $$\frac{\partial {{{{{\bf{B}}}}}}}{\partial t}+{{{{{\boldsymbol{\nabla }}}}}}\cdot \left({{{{{\bf{VB}}}}}}-{{{{{\bf{BV}}}}}}\right)=\eta {\nabla }^{2}{{{{{\bf{B}}}}}},$$

9 $$e=\frac{1}{2}\rho {V}^{2}+\frac{p}{\gamma -1}+\frac{1}{2}{B}^{2},$$

10 $${{{{{\bf{j}}}}}}={{{{{\boldsymbol{\nabla }}}}}}\times {{{{{\bf{B}}}}}}.$$

$e$ and ${{{{{\bf{j}}}}}}$ correspond to the total energy density and current density, respectively. $\rho$ is the mass density, ${{{{{\bf{V}}}}}}$ is the flow velocity, ${P}_{{{{{{\rm{th}}}}}}}$ is the thermal pressure, ${{{{{\bf{B}}}}}}$ denotes the magnetic field, $\gamma \left(=5/3\right)$ is the adiabatic index, $\nu \left(={10}^{-4}\right)$ is the viscosity, $\eta (={10}^{-4})$ is the magnetic resistivity, and $t$ is the time. To solve these equations in a cube with a side length of 2*π*, we employ the higher-order Godunov code Athena^73^. Specifically, we apply a third-order piecewise parabolic method (PPM) to the reconstruction, the approximate Riemann solver with Harten–Lax–van Leer discontinuities (HLLD) to the calculation of the numerical fluxes, and the constrained transport algorithm for ensuring the divergence-free state of the magnetic field. Each side of the cubical grid of edge length $L$ is discretized by 1024 uniform grid points, and we implement periodic boundary conditions.

We initialize our simulation with non-compressive waves, and a background magnetic field ${{{{{{\bf{B}}}}}}}_{0}$ (=1) is imposed along the *z-*direction. The magnetic field can be written as ${{{{{{\bf{B}}}}}}{{{{{\boldsymbol{=}}}}}}{{{{{\bf{B}}}}}}}_{0}+{{{{{\rm{\delta }}}}}}{{{{{\bf{B}}}}}}$, with ${{{{{\rm{\delta }}}}}}{{{{{\bf{B}}}}}}$ being the fluctuating magnetic field. The initial velocity and magnetic field fluctuations ${{{{{\rm{\delta }}}}}}{{{{{\bf{V}}}}}}$ and ${{{{{\rm{\delta }}}}}}{{{{{\bf{B}}}}}}$ populate a shell in Fourier $k-$ space with $2\le k\le 5$. We set these modes so that they have constant amplitude and random phases. The initial root-mean-square of ${{{{{\rm{\delta }}}}}}{{{{{\bf{V}}}}}}$ and ${{{{{\rm{\delta }}}}}}{{{{{\bf{B}}}}}}$ is about 1, resulting in an initial turbulent Mach number and initial Alfvén Mach number of about 1.1 and 1, respectively. We run the model of decaying MHD turbulence to approach a state when the power-law spectrum is fully developed. The simulation run lasts about 6 Alfvén times ${t}_{{V}_{A}}=1/({{{{{{\rm{B}}}}}}}_{0}/\sqrt{{{{{{{\rm{\rho }}}}}}}_{0}})$, at which time ${ < {{{{{\rm{|}}}}}}{{{{{\rm{\delta }}}}}}{{{{{{\rm{B}}}}}}}_{x}^{2}{{{{{\rm{|}}}}}} > }^{0.5}/{B}_{0}$ drops to about 0.5. Our simulation traces the evolution with time of the fluctuating kinetic energy, the fluctuating magnetic energy, the fluctuating Elsässer variables, and the normalized cross-helicity (Supplementary Fig. 3).

### Computation of the auto-correlation function

We calculate the auto-correlation function (ACF) of ${{{{{\rm{\delta }}}}}}{{{\rm {Z}}}}_{x}^{+}$ and ${{{{{\rm{\delta }}}}}}{{{{{{\rm{Z}}}}}}}_{x}^{-}$ as

11 $${{{{{{\rm{ACF}}}}}}}_{{{{{{\rm{\delta }}}}}}{{{\rm {Z}}}}_{x}^{\pm }}\left({{{{{\rm{l}}}}}},{{{{{\rm{\tau }}}}}}\right)=\iint {{{{{{\rm{\delta }}}}}}{{\rm {Z}}}}_{x}^{\pm }(z,t){{{{{{\rm{\delta }}}}}}{{\rm {Z}}}}_{x}^{\pm }(z+l,t+\tau )\, {{{{{\rm{d}}}}}}z\, {{{{{\rm{d}}}}}}t,$$

with *l* being the spatial lag and $\tau$ being the time lag.

### Comparison of large-scale and local-sale energy transfer rates

To investigate energy transfer across different scales, we represent the fluctuating Elsässer variables ${{{{{{\rm{\delta }}}}}}{{{{{\bf{Z}}}}}}}^{\pm }$ by their Fourier series expansions, ${{{{{{\rm{\delta }}}}}}{{{{{\bf{Z}}}}}}}^{\pm }={\sum }_{{{{{{\bf{k}}}}}}}{{{{{\rm{\delta }}}}}}{\widetilde{{{{{{\rm{Z}}}}}}}}^{\pm }({{{{{\bf{k}}}}}}){{{{{{\rm{e}}}}}}}^{{{{{{\rm{i}}}}}}{{{{{\bf{k}}}}}}\cdot {{{{{\bf{x}}}}}}}$. We then divide the wavenumber space into spherical shells of unit width centered around the origin. Preserving only the modes in Fourier space with wavenumbers satisfying $K\le \left|{{{{{\bf{K}}}}}}\right| < K+1$, where $K$ is the shell radius, yields the filtered fields, which are denoted as ${{{{{{\rm{\delta }}}}}}{{{{{\bf{Z}}}}}}}_{K}^{\pm }$. For ${{{{{{\rm{\delta }}}}}}{{{{{\bf{Z}}}}}}}^{\pm }$, the rate of energy transfer $\varepsilon (K,Q,P)$ from energy in shell $Q$ to energy in shell $K$ due to the interaction with ${{{{{{\rm{\delta }}}}}}{{{{{\bf{Z}}}}}}}^{{{\mp }}}$ in shell $P$ is then

12 $${\varepsilon (K,Q,P)}^{{{{{{{\rm{\delta }}}}}}{{{{{\rm{Z}}}}}}}^{{{\pm }}}}=\int {{{{{\rm{\delta }}}}}}{{{{{{\bf{Z}}}}}}}_{K}^{\pm }\cdot ({{{{{\rm{\delta }}}}}}{{{{{{\bf{Z}}}}}}}_{P}^{\mp }\cdot {{{{{\boldsymbol{\nabla }}}}}}){{{{{\rm{\delta }}}}}}{{{{{{\bf{Z}}}}}}}_{Q}^{\pm }{{{{{{\rm{d}}}}}}}^{3}x.$$

In order to quantify the contributions of scale non-locality and scale locality, we calculate the ratio of the transfer rate due to the large-scale interactions to the total transfer rate, denoted as ${\varepsilon }_{{{{{{\rm{LS}}}}}},{{{{{\rm{b}}}}}}}^{{{{{{{\rm{\delta }}}}}}{\rm {{Z}}}}^{{{\pm }}}}/{\varepsilon }_{{{{{{\rm{total}}}}}},{{{{{\rm{b}}}}}}}^{{{{{{{\rm{\delta }}}}}}{{\rm {Z}}}}^{{{\pm }}}}$, on a band of $K$-shells between ${K}_{\min }=K/\sqrt{2}$ and $K=\sqrt{2}K$ as

13 $$\frac{{\varepsilon }_{{{{{{\rm{LS}}}}}}}^{{{{{{{\rm{\delta }}}}}}{{\rm {Z}}}}^{{{\pm }}}}}{{\varepsilon }_{{{{{{\rm{total}}}}}}}^{{{{{{{\rm{\delta }}}}}}{{\rm {Z}}}}^{{{\pm }}}}}=\mathop{\sum }\limits_{{K}_{\min }}^{{K}_{\max }}\mathop{\sum }\limits_{Q=1}^{K-1}{{{{{{\rm{\varepsilon }}}}}}}_{{P} < 6}^{{{{{{{\rm{\delta }}}}}}{{\rm {Z}}}}^{{{\pm }}}}/\mathop{\sum }\limits_{{K}_{\min }}^{{K}_{\max }}\mathop{\sum }\limits_{Q=1}^{K-1}{{{{{{\rm{\varepsilon }}}}}}}_{{{\rm {all}}\; P}}^{{{{{{{\rm{\delta }}}}}}{{\rm {Z}}}}^{{{\pm }}}},$$

and the ratio of the transfer rate due to local-scale interactions to the total transfer rate, ${\varepsilon }_{{{{{{\rm{local}}}}}},{{{{{\rm{b}}}}}}}^{{{{{{{\rm{\delta }}}}}}{{{{{\rm{Z}}}}}}}^{{{\pm }}}}/{\varepsilon }_{{{{{{\rm{total}}}}}},{{{{{\rm{b}}}}}}}^{{{{{{{\rm{\delta }}}}}}{{{{{\rm{Z}}}}}}}^{{{\pm }}}}$, as

14 $$\frac{{\varepsilon }_{{{{{{\rm{local}}}}}}}^{{{{{{{\rm{\delta }}}}}}{{{{{\rm{Z}}}}}}}^{{{\pm }}}}}{{\varepsilon }_{{{{{{\rm{total}}}}}}}^{{{{{{{\rm{\delta }}}}}}{{{{{\rm{Z}}}}}}}^{{{\pm }}}}}=\mathop{\sum }\limits_{{K}_{\min }}^{{K}_{\max }}\mathop{\sum }\limits_{Q=1}^{K-1}{{{{{{\rm{\varepsilon }}}}}}}_{{{{{{\rm{local\; P}}}}}}}^{{{{{{{\rm{\delta }}}}}}{{{{{\rm{Z}}}}}}}^{{{\pm }}}}/\mathop{\sum }\limits_{{K}_{\min }}^{{K}_{\max }}\mathop{\sum }\limits_{Q=1}^{K-1}{{{{{{\rm{\varepsilon }}}}}}}_{{{{{{\rm{all\; P}}}}}}}^{{{{{{{\rm{\delta }}}}}}{{{{{\rm{Z}}}}}}}^{{{\pm }}}}.$$

Here, the total transfer rate ${\varepsilon }_{{{{{{\rm{all\; P}}}}}}}^{{{{{{{\rm{\delta }}}}}}{{{{{\rm{Z}}}}}}}^{{{\pm }}}}$, the transfer rate due to the work by large-scale fields ${\varepsilon }_{{P} < 6}^{{{{{{{\rm{\delta }}}}}}{Z}}^{{{\pm }}}}$, and the transfer rate due to local-scale interactions ${\varepsilon }_{{{{{{\rm{local}}}}}}\; P}^{{{{{{{\rm{\delta }}}}}}{{\rm {Z}}}}^{{{\pm }}}}$ are obtained by $\mathop{\sum }\limits_{P=1}^{{P}_{\max }}{\varepsilon (K,Q,P)}^{{{{{{{\rm{\delta }}}}}}{{{{{\rm{Z}}}}}}}^{{{\pm }}}}$, $\mathop{\sum }\limits_{P=1}^{P=5}{\varepsilon (K,Q,P)}^{{{{{{{\rm{\delta }}}}}}{{\rm {Z}}}}^{{{\pm }}}}$, and $\mathop{\sum }\limits_{P=Q/2}^{P=2Q}{\varepsilon (K,Q,P)}^{{{{{{{\rm{\delta }}}}}}{{\rm {Z}}}}^{{{\pm }}}}$, respectively. Consistent with the works by Mininni et al.^74^, the $P$ modes <6 are considered responsible for large-scale interactions. This choice is also consistent with our simulation for which we initially impose large-scale fluctuations with $P$ < 6. The collection of *P* modes that lead to the occurrence of the local-scale interactions agrees with Alexakis et al. ^51^. The band of *K*-shells is consistent with that used by Cho et al. ^13^.

### Supplementary information

Supplementary Information

Description of Additional Supplementary Files

Supplementary Movie 1

Supplementary Movie 2

## Data Availability

The calibrated observational data from the PSP spacecraft is available from the CDAWeb website (https://cdaweb.gsfc.nasa.gov/) or from the FTP server (ftp://spdf.gsfc.nasa.gov/). It is worth noting that both of these two platforms offer identical datasets. The data directly used for the display figures can be accessed at https://figshare.com under 10.6084/m9.figshare.24064473. The datasets generated and analyzed during the current study are available from the corresponding author upon request.

## Code availability

The PSP data were analyzed with the PySpedas, which can be downloaded from https://github.com/spedas/pyspedas, and installed through “pip install pyspedas” in the python environment. The simulations of the compressible MHD turbulence were conducted with the code Athena, which can be downloaded from https://princetonuniversity.github.io/Athena-Cversion/. The RMHD turbulence was modeled by using the code rmhdtod, which is available from https://sourceforge.net/projects/rmhdtod/. The analysis of the simulation data can be done with any science program language (e.g., Python) according to the formulas provided explicitly in the manuscript.
