# Supplementary material for: Energy transfer of imbalanced Alfvénic turbulence in the heliosphere
Source: Nat Commun. 2023 Dec 2;14:7955. doi: 10.1038/s41467-023-43273-4 (PMC10692179; doi:10.1038/s41467-023-43273-4)
Supplement: Supplementary file 1 — Supplementary Information [file 41467_2023_43273_MOESM1_ESM.pdf]

Supplementary Materials for

Energy Transfer of Imbalanced Alfvénic Turbulence in the Heliosphere

Liping Yang, Jiansen He, Daniel Verscharen, Hui Li, Trevor A. Bowen, Stuart D. Bale, Honghong Wu, Wenya Li, Ying Wang, Lei Zhang, Xueshang Feng, Ziqi Wu

Correspondence to: jshept@pku.edu.cn

This PDF file includes:

Supplementary Methods
Supplementary Discussion
Supplementary Fig. 1 to 13
Supplementary References

Supplementary Methods

Equations of the fluctuating Elsässer variables

From the MHD equations, a set of the Elsässer equations can be derived[1]:

$$\frac{\partial \mathbf{Z}^+}{\partial t} + (\mathbf{Z}^- \cdot \nabla)\mathbf{Z}^+ + \frac{1}{\rho}\nabla P_t + \frac{\mathbf{V}_A}{2}\nabla \cdot (\mathbf{Z}^+ + \mathbf{V}_A) = 0, \quad (1)$$

$$\frac{\partial \mathbf{Z}^-}{\partial t} + (\mathbf{Z}^+ \cdot \nabla)\mathbf{Z}^- + \frac{1}{\rho}\nabla P_t - \frac{\mathbf{V}_A}{2}\nabla \cdot (\mathbf{Z}^- - \mathbf{V}_A) = 0, \quad (2)$$

where $\mathbf{Z}^+ = \mathbf{V} + \mathbf{V}_A, \mathbf{Z}^- = \mathbf{V} - \mathbf{V}_A$ $\mathbf{V}_A = \frac{\mathbf{B}}{\sqrt{\rho}}$, and P_t is total pressure. We define the fluctuating Elsässer variables as: $\delta\mathbf{Z}^+ = \delta\mathbf{V} + \delta\mathbf{V}_A, \delta\mathbf{Z}^- = \delta\mathbf{V} - \delta\mathbf{V}_A$, with $\delta\mathbf{V}_A = \frac{\delta\mathbf{B}}{\sqrt{\rho}}$, $\mathbf{V}_{A0} = \frac{\mathbf{B}_0}{\sqrt{\rho}}$, so that $\mathbf{Z}^+ = \delta\mathbf{Z}^+ + \mathbf{V}_{A0}, \mathbf{Z}^- = \delta\mathbf{Z}^- - \mathbf{V}_{A0}$. Then, (1) and (2) can be re-written as:

$$\frac{\partial \delta\mathbf{Z}^+}{\partial t} + \frac{\partial \mathbf{V}_{A0}}{\partial t} + [(\delta\mathbf{Z}^- - \mathbf{V}_{A0}) \cdot \nabla](\delta\mathbf{Z}^+ + \mathbf{V}_{A0}) + \frac{1}{\rho}\nabla P_t +$$
$$\frac{1}{2}(\frac{\delta\mathbf{Z}^+ - \delta\mathbf{Z}^-}{2} + \mathbf{V}_{A0})\nabla \cdot \left(\delta\mathbf{Z}^+ + \mathbf{V}_{A0} + \frac{\delta\mathbf{Z}^+ - \delta\mathbf{Z}^-}{2} + \mathbf{V}_{A0}\right) = 0, \quad (3)$$

$$\frac{\partial \delta\mathbf{Z}^-}{\partial t} - \frac{\partial \mathbf{V}_{A0}}{\partial t} + [(\delta\mathbf{Z}^+ + \mathbf{V}_{A0}) \cdot \nabla](\delta\mathbf{Z}^- - \mathbf{V}_{A0}) + \frac{1}{\rho}\nabla P_t -$$
$$\frac{1}{2}(\frac{\delta\mathbf{Z}^+ - \delta\mathbf{Z}^-}{2} + \mathbf{V}_{A0})\nabla \cdot \left(\delta\mathbf{Z}^- - \mathbf{V}_{A0} - \frac{\delta\mathbf{Z}^+ - \delta\mathbf{Z}^-}{2} - \mathbf{V}_{A0}\right) = 0, \quad (4)$$

As $\mathbf{V}_{A0} = \frac{\mathbf{B}_0}{\sqrt{\rho}}$, the direction of $\mathbf{V}_{A0}$ is in the direction parallel to $\mathbf{B_0}$. When deriving the equations of the perpendicular fluctuating Elsässer variables ($\delta\mathbf{Z}_\perp^\pm$), we can reasonably assume zero values of $\frac{\partial \mathbf{V}_{A0}}{\partial t}$, $\left[\left(\delta\mathbf{Z}^\mp \mp \mathbf{V}_{A0}\right) \cdot \nabla\right]\mathbf{V}_{A0}$, and $\mathbf{V}_{A0}\nabla \cdot \left(\delta\mathbf{Z}^\pm \pm \mathbf{V}_{A0} \pm \frac{\delta\mathbf{Z}^+ - \delta\mathbf{Z}^-}{2} \pm \mathbf{V}_{A0}\right)$ since all of these terms are along the parallel direction and thus only contribute to the variations of the parallel fluctuating Elsässer variables. Under these assumptions, we obtain the equations of the fluctuating Elsässer variables perpendicular to $\mathbf{B}_0$:

$$\frac{\partial \delta\mathbf{Z}_\perp^+}{\partial t} - (\mathbf{V}_{A0} \cdot \nabla)\delta\mathbf{Z}_\perp^+ + (\delta\mathbf{Z}^- \cdot \nabla)\delta\mathbf{Z}_\perp^+ + \frac{1}{\rho}\nabla P_t +$$
$$\frac{\delta\mathbf{Z}_\perp^+ - \delta\mathbf{Z}_\perp^-}{8}\nabla \cdot (3\delta\mathbf{Z}^+ - \delta\mathbf{Z}^-) + \frac{\delta\mathbf{Z}_\perp^+ - \delta\mathbf{Z}_\perp^-}{2}\nabla \cdot \mathbf{V}_{A0} = 0, \quad (5)$$

$$\frac{\partial \delta\mathbf{Z}_\perp^-}{\partial t} + (\mathbf{V}_{A0} \cdot \nabla)\delta\mathbf{Z}_\perp^- + (\delta\mathbf{Z}^+ \cdot \nabla)\delta\mathbf{Z}_\perp^- + \frac{1}{\rho}\nabla P_t -$$
$$\frac{\delta\mathbf{Z}_\perp^+ - \delta\mathbf{Z}_\perp^-}{8}\nabla \cdot (3\delta\mathbf{Z}^- - \delta\mathbf{Z}^+) + \frac{\delta\mathbf{Z}_\perp^+ - \delta\mathbf{Z}_\perp^-}{2}\nabla \cdot \mathbf{V}_{A0} = 0, \quad (6)$$

Comparing to the incompressible equations for $\delta\mathbf{Z}_\perp^\pm$, the compressible equations for $\delta\mathbf{Z}_\perp^\pm$ in (5)-(6) have two additional terms: one relates to the compressibility of $\delta\mathbf{Z}^\pm$, $\pm\frac{\delta\mathbf{Z}_\perp^+ - \delta\mathbf{Z}_\perp^-}{8}\nabla \cdot \left(3\delta\mathbf{Z}^\pm - \delta\mathbf{Z}^\mp\right)$, and the other relates to density fluctuations, $\frac{\delta\mathbf{Z}_\perp^+ - \delta\mathbf{Z}_\perp^-}{2}\nabla \cdot \mathbf{V}_{A0}$. These two terms modify the interpretation of the Elsässer variables compared to the incompressible case: they no longer represent fluctuations with opposite directions of propagation, nor can they be considered as merely counter-propagating fluctuations. According to Equations (5-6), in the compressible

regime, nonlinearities do not only arise from counter-propagating fluctuations $\delta \mathbf{Z}_{\perp}^{\pm}$. Besides $(\delta \mathbf{Z}^{\mp} \cdot \nabla)\delta \mathbf{Z}_{\perp}^{\pm}$, the compressibility terms $\pm \frac{\delta Z_{\perp}^{+} - \delta Z_{\perp}^{-}}{8} \nabla \cdot \left(3\delta \mathbf{Z}^{\pm} - \delta \mathbf{Z}^{\mp}\right)$ also contribute to nonlinearities.

Based on Equations (5)-(6), we analyze the contributions of the linear terms ($\pm(\mathbf{V}_{\mathrm{A0}} \cdot \nabla)\delta \mathbf{Z}_{\perp}^{\pm}$), the nonlinear terms ($-(\delta \mathbf{Z}^{\mp} \cdot \nabla)\delta \mathbf{Z}_{\perp}^{\pm}$), and the generalized compressible terms $T_{\mathrm{comp}}^{\delta \mathbf{Z}_{\perp}^{\pm}} = -\frac{1}{\rho}\nabla P_t \mp \frac{\delta Z_{\perp}^{+} - \delta Z_{\perp}^{-}}{8} \nabla \cdot \left(3\delta \mathbf{Z}^{\pm} - \delta \mathbf{Z}^{\mp}\right) - \frac{\delta Z_{\perp}^{+} - \delta Z_{\perp}^{-}}{2} \nabla \cdot \mathbf{V}_{\mathrm{A0}}$ to the evolution of the perpendicular fluctuating Elsässer variables ($\frac{\partial \delta \mathbf{Z}_{\perp}^{\pm}}{\partial t}$) as well as the scale locality of nonlinear interactions. From the numerical solution of the MHD equations, we discretize ∇ and $\frac{\partial}{\partial t}$ in (5) and (6) through central differences to obtain the spatial distributions of $\frac{\partial \delta \mathbf{Z}_{\perp}^{\pm}}{\partial t}$, $\pm(\mathbf{V}_{\mathrm{A0}} \cdot \nabla)\delta \mathbf{Z}_{\perp}^{\pm}$, $-(\delta \mathbf{Z}^{\mp} \cdot \nabla)\delta \mathbf{Z}_{\perp}^{\pm}$, as well as $T_{\mathrm{comp}}^{\delta \mathbf{Z}_{\perp}^{\pm}}$.

Fig. S3 illustrates the time evolution of the fluctuating kinetic energy, the fluctuating magnetic energy, the fluctuating Elsässer variables ($\delta \mathbf{Z}^{+}$ and $\delta \mathbf{Z}^{-}$), and the normalized cross-helicity σ_c for the case with an initial cross-helicity of 0.7.

Fig. S8 presents 2D distributions of the discretized $\pm(\mathbf{V_{A0}} \cdot \nabla)\delta Z_x^{\pm}$, $-\left(\boldsymbol{\delta} \mathbf{Z}^{\mp} \cdot \nabla\right)\delta Z_x^{\pm}, T_{\mathrm{com}}^{\delta Z_x^{\pm}}$, and their PSD as functions of wavenumber k but for the case with an initial cross-helicity of 0. At $k > 5$, the PSDs of the linear and nonlinear terms are approximately equal, suggesting the critical balance between the linear and nonlinear times.

Supplementary Discussion

Estimation of the spectral indexes for $\delta \mathbf{Z}^{+}$ and $\delta \mathbf{Z}^{-}$

For $\delta \mathbf{Z}^{-}$, the large-scale fields of $\delta \mathbf{Z}^{+}$ influence the energy cascade of $\delta \mathbf{Z}^{-}$ more than the local-scale fields. We estimate $\delta \mathbf{Z}^{-}$'s nonlinear time as $\tau_{\delta Z^{-}} \sim L/\delta Z_{\mathrm{L}}^{+}$, with L being the large scale. From the constant energy flux of $\delta \mathbf{Z}^{-}$, $\epsilon_{\delta Z^{-}} \sim \frac{|\delta Z^{-}|^2}{\tau_{\delta Z^{-}}}$, it can be derived that $|\delta \mathbf{Z}^{-}|^2$ does not change with scale, leading to an energy spectrum $\sim k^{-1}$.

From the fluctuating Elsässer equations (5-6), the nonlinear term for the evolution of $\delta \mathbf{Z}^{+}$is $\delta \mathbf{Z}^{-} \cdot \nabla \delta \mathbf{Z}^{+} \sim \delta \mathbf{Z}^{-} \cdot \mathbf{k}_{\perp}^{+} \delta \mathbf{Z}^{+} \sim \delta Z^{-} k_{\perp}^{+} \cos\theta_{\delta Z^{-} k_{\perp}^{+}} \delta Z^{+}$, where $\theta_{\delta Z^{-} k_{\perp}^{+}}$ is the angle between $\delta \mathbf{Z}^{-}$ and $\mathbf{k}_{\perp}^{+}$. The nonlinear time for $\delta \mathbf{Z}^{+}$ can be estimated as $\tau_{\delta Z^{+}} \sim (\delta Z^{-} k_{\perp}^{+} \cos\theta_{\delta Z^{-} k_{\perp}^{+}})^{-1}$. Since δZ^{-} shows no variations with scale, $\tau_{\delta Z^{+}} \sim (k_{\perp}^{+} \cos\theta_{\delta Z^{-} k_{\perp}^{+}})^{-1}$. The variation of $\cos\theta_{\delta Z^{-} k_{\perp}^{+}}$ with scale influences the spectral index of $\delta \mathbf{Z}^{+}$.

If we assume that $\cos\theta_{\delta Z^{-} k_{\perp}^{+}}$ does not depend on scale, $\tau_{\delta Z^{+}} \sim k_{\perp}^{+\,-1}$. Under the condition of the constant energy flux, an energy spectrum $\sim k^{-2}$ is obtained for $\delta \mathbf{Z}^{+}$.

If the phenomenon of scale-dependent dynamic alignment occurs, the alignment angle varies with scale as $\theta_{\delta Z^{+} \delta Z^{-}} \sim k^{-1/4}$(2). Since $\delta \mathbf{Z}^{+}$, to first order, behaves like Alfvén waves, $\delta \mathbf{Z}^{+} \perp \mathbf{k}_{\perp}^{+}$, $\cos\theta_{\delta Z^{-} k_{\perp}^{+}} = \sin\theta_{\delta Z^{+} \delta Z^{-}}$. Then,

$\tau_{\delta Z^+} \sim (k_\perp^+ \cos\theta_{\delta Z^- k_\perp^+})^{-1} \sim (k_\perp^+ \sin\theta_{\delta Z^+ \delta Z^-})^{-1} \sim (k_\perp^+ \theta_{\delta Z^+ \delta Z^-})^{-1} \sim {k_\perp^+}^{-3/4}$. The energy flux of $\delta\mathbf{Z}^+$ is estimated as $\epsilon_{\delta Z^+} \sim \frac{|\delta \mathbf{Z}^+|^2}{\tau_{\delta Z^+}} \sim |\delta\mathbf{Z}^+|^2 {k_\perp^+}^{3/4}$, which results in an energy spectrum $\sim k^{-1.75}$ for $\delta\mathbf{Z}^+$.

If the angle varies with scale as $\theta_{\delta Z^+ \delta Z^-} \sim k^{-1/3}$, $\tau_{\delta Z^+} \sim {k_\perp^+}^{-2/3}$, and $\delta\mathbf{Z}^+$ would have an energy spectrum $\sim k^{-5/3}$.

Fig. S12 shows the probability density function (PDF) of linear time over nonlinear time for δZ_x^+ and δZ_x^-, with the linear and nonlinear time being estimated by the discretized terms $\left|\frac{\delta Z_x^\pm}{(\mathbf{V_{A0}}\cdot\nabla)\delta Z_x^\pm}\right|$ and $\left|\frac{\delta Z_x^\pm}{(\boldsymbol{\delta Z^\mp}\cdot\nabla)\delta Z_x^\pm}\right|$, respectively. For δZ_x^+, the linear time is less than the nonlinear time, while for δZ_x^-, the linear time is greater than the nonlinear time.

Supplementary Fig. 1-13

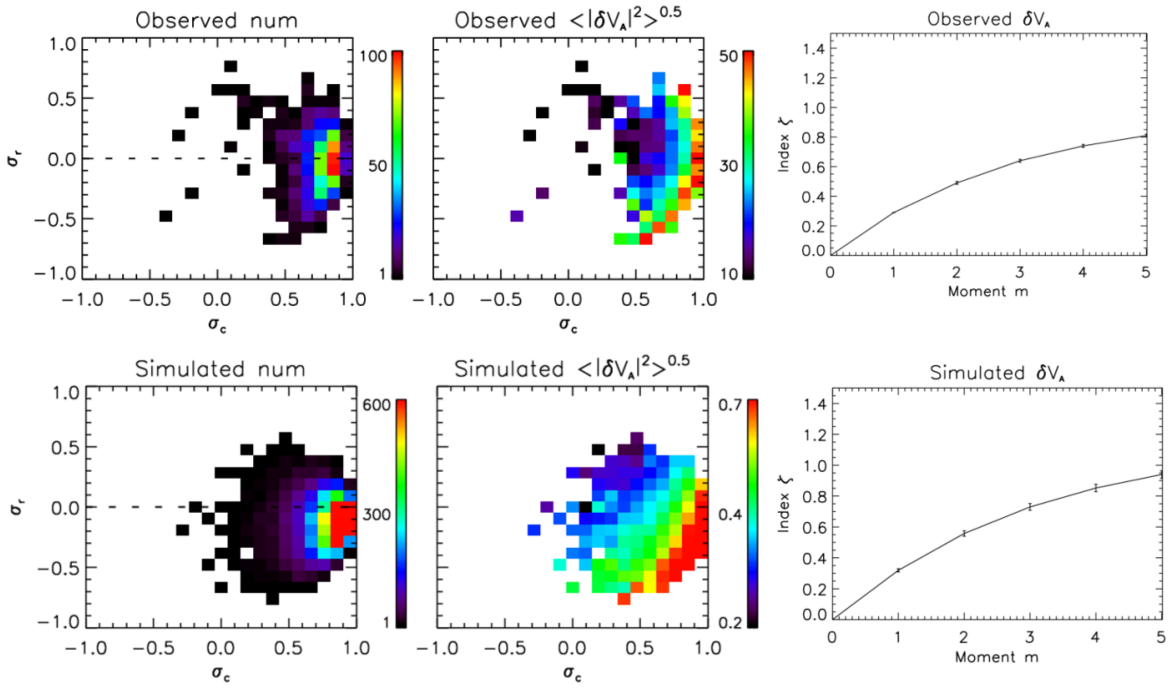

Supplementary Fig. 1 Distributions of occurrence numbers and pixel-averaged $< |\delta \mathbf{V}_A^2| >^{0.5}$ in the $\sigma_r - \sigma_c$ plane as well as variations of the intermittency-related scaling exponents with the order moments of the structure function for the PSP data when the PSP was around its first perihelion (upper panels) and for the numerical simulation data at time = 4.0 for the case with an initial cross-helicity of 0.7 (lower panels).

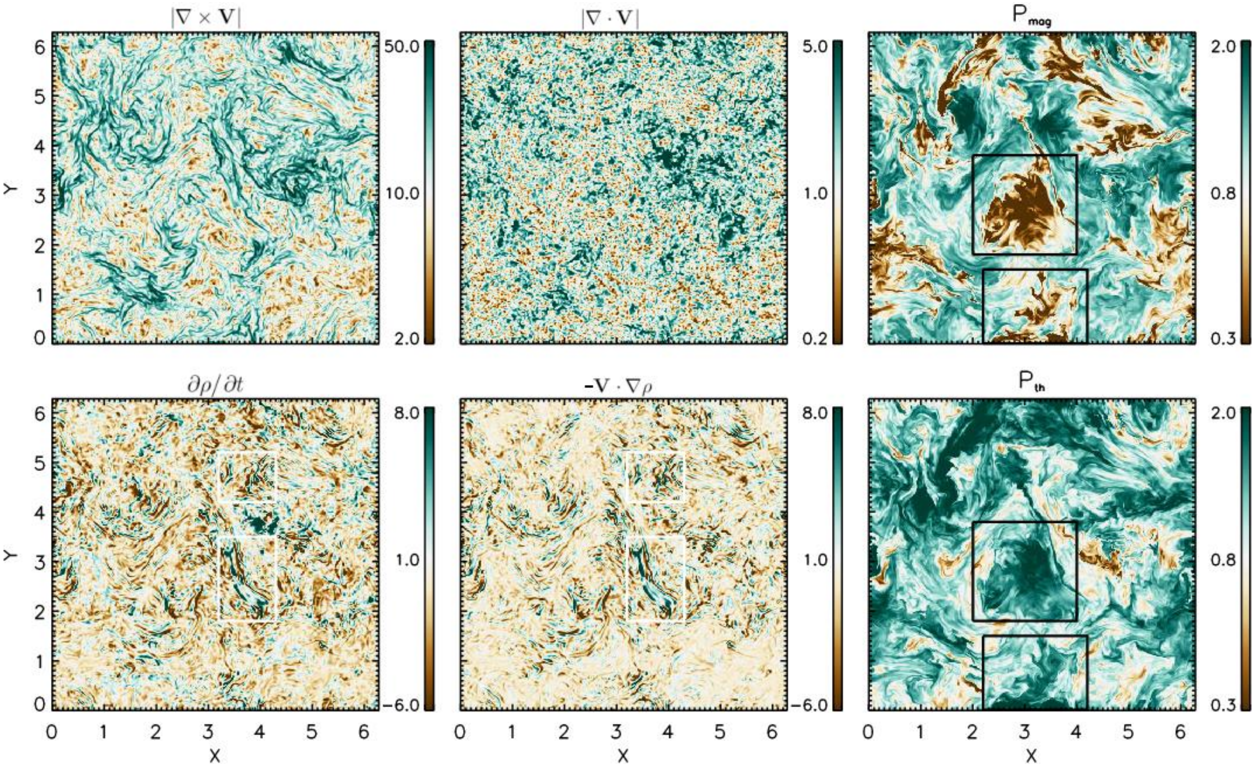

Supplementary Fig. 2 2D distributions of the discretized $|\boldsymbol{\nabla} \times \mathbf{V}|$, $|\boldsymbol{\nabla} \cdot \mathbf{V}|$, $\partial\rho/\partial t$, $-\mathbf{V} \cdot \boldsymbol{\nabla}\rho$, magnetic pressure P_{mag}, and thermal pressure P_{th} at time $= 4.0$ for the case with an initial cross-helicity of 0.7.

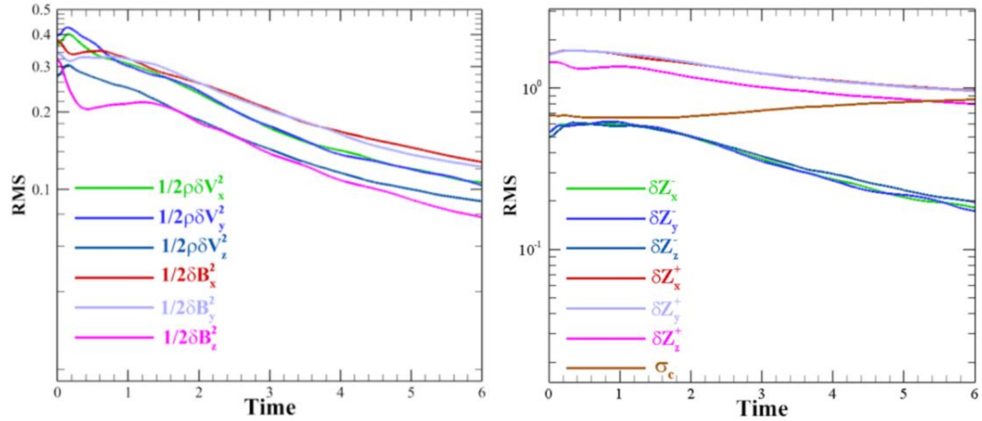

Supplementary Fig. 3 Time evolution of the fluctuating kinetic energy ($\frac{1}{2}\rho\delta V_x^2$, $\frac{1}{2}\rho\delta V_y^2$, $\frac{1}{2}\rho\delta V_z^2$), the fluctuating magnetic energy ($\frac{1}{2}\delta B_x^2$, $\frac{1}{2}\delta B_y^2$, $\frac{1}{2}\delta B_z^2$), the fluctuating Elsässer variables ($\delta\mathbf{Z}^+$ and $\delta\mathbf{Z}^-$) – all shown component by component, and the normalized cross-helicity σ_c for the case with an initial cross-helicity of 0.7.

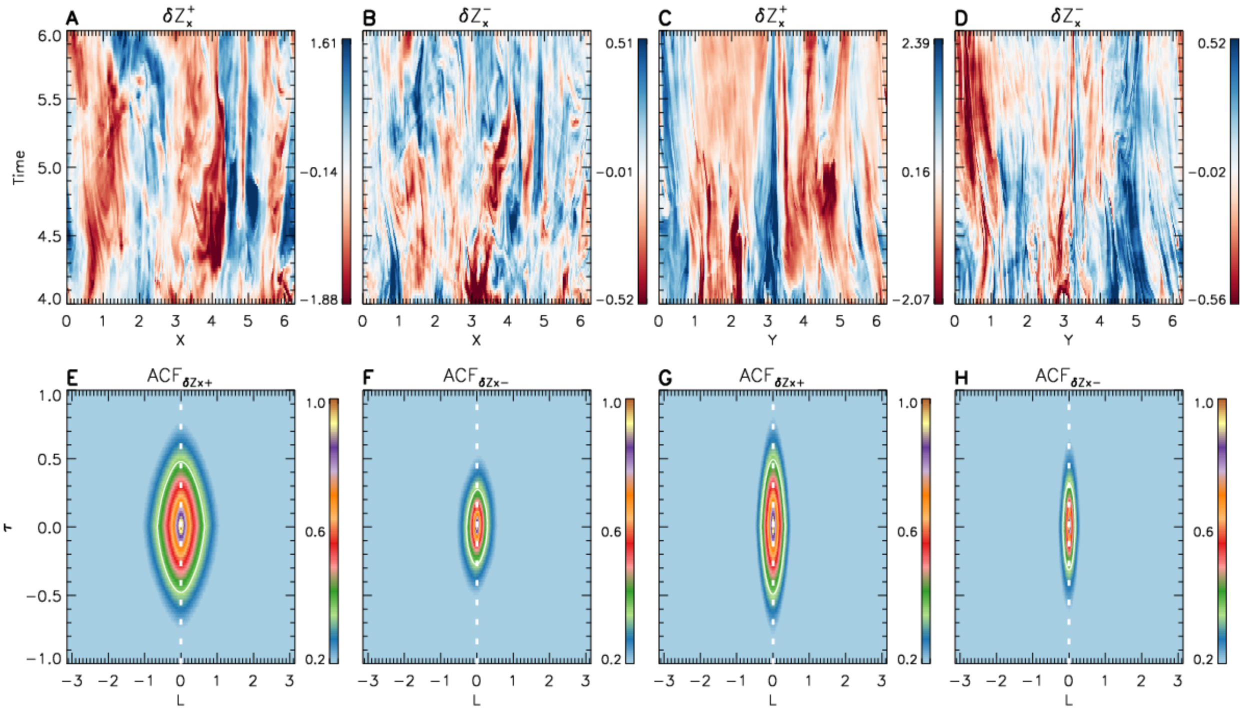

Supplementary Fig. 4 Propagation of δZ_x^+ and δZ_x^- along perpendicular directions for the case with an initial cross-helicity of 0.7. (**A, B, C** and **D**) Time-distance diagrams of δZ_x^+ and δZ_x^- for the time from 4.0 until 6.0 for the 1D cut along the x −direction (**A** and **B**) and for the 1D cut along the y −direction (**C** and **D**). (**E, F, G** and **H**) Distributions of the auto-correlation function (ACF) of δZ_x^+ and δZ_x^- in the τ (time lag) $-l$ (spatial lag) plane for the 1D cut along the x −direction (**E** and **F**) and for the 1D cut along the y −direction (**G** and **H**). The white dashed lines are along $l = 0$.

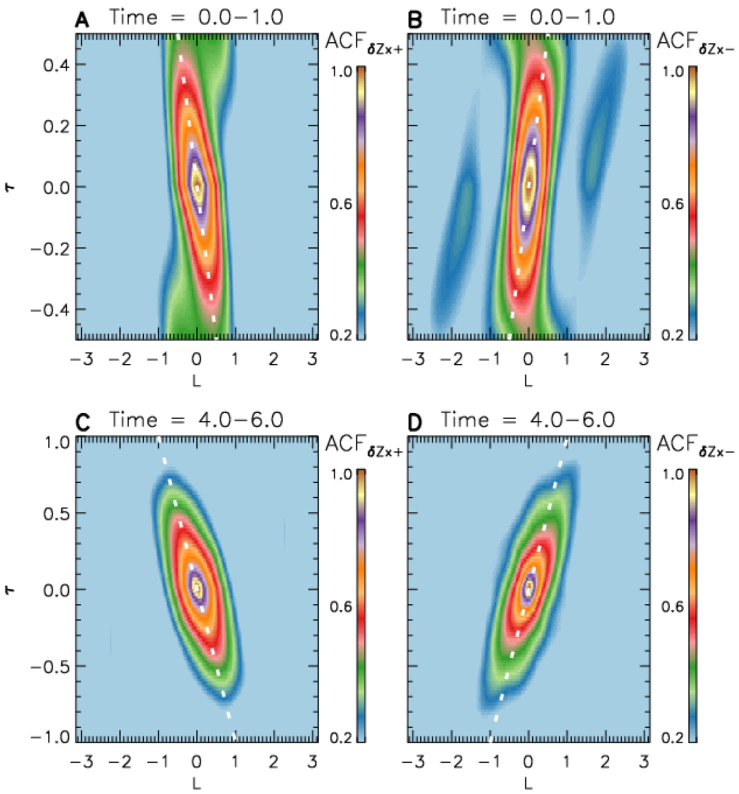

Supplementary Fig. 5 Distributions of the auto-correlation function (ACF) of δZ_x^+ and δZ_x^- in the τ (time lag) $-l$ (spatial lag in the z −direction) plane for the time from 0.0 until1.0 (**A** and **B**) and from 4.0 until 6.0 (**C** and **D**) for the case with an initial cross-helicity of 0.0. The white dashed lines show the expected propagation of Alfvén waves along the z −direction.

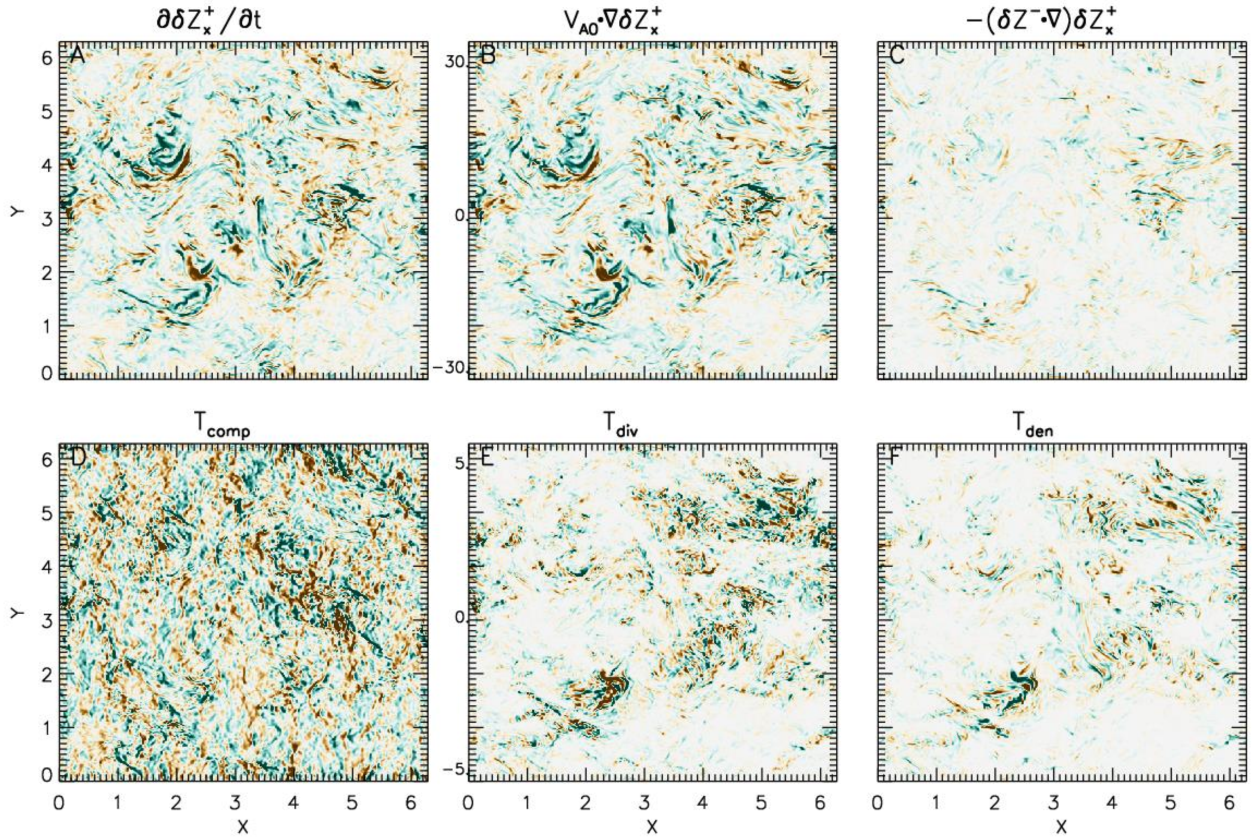

Supplementary Fig. 6 Distributions of the terms in the governing equation of the perpendicular fluctuating Elsässer variable δZ_x^+ at time $= 4.0$ for the case with an initial cross-helicity of 0.7. (A) the evolution term, (B) the linear term, (C) the nonlinear term , (D) the generalized compressible term T_{comp}, (E) the term T_{div}, (F) the term T_{den}.

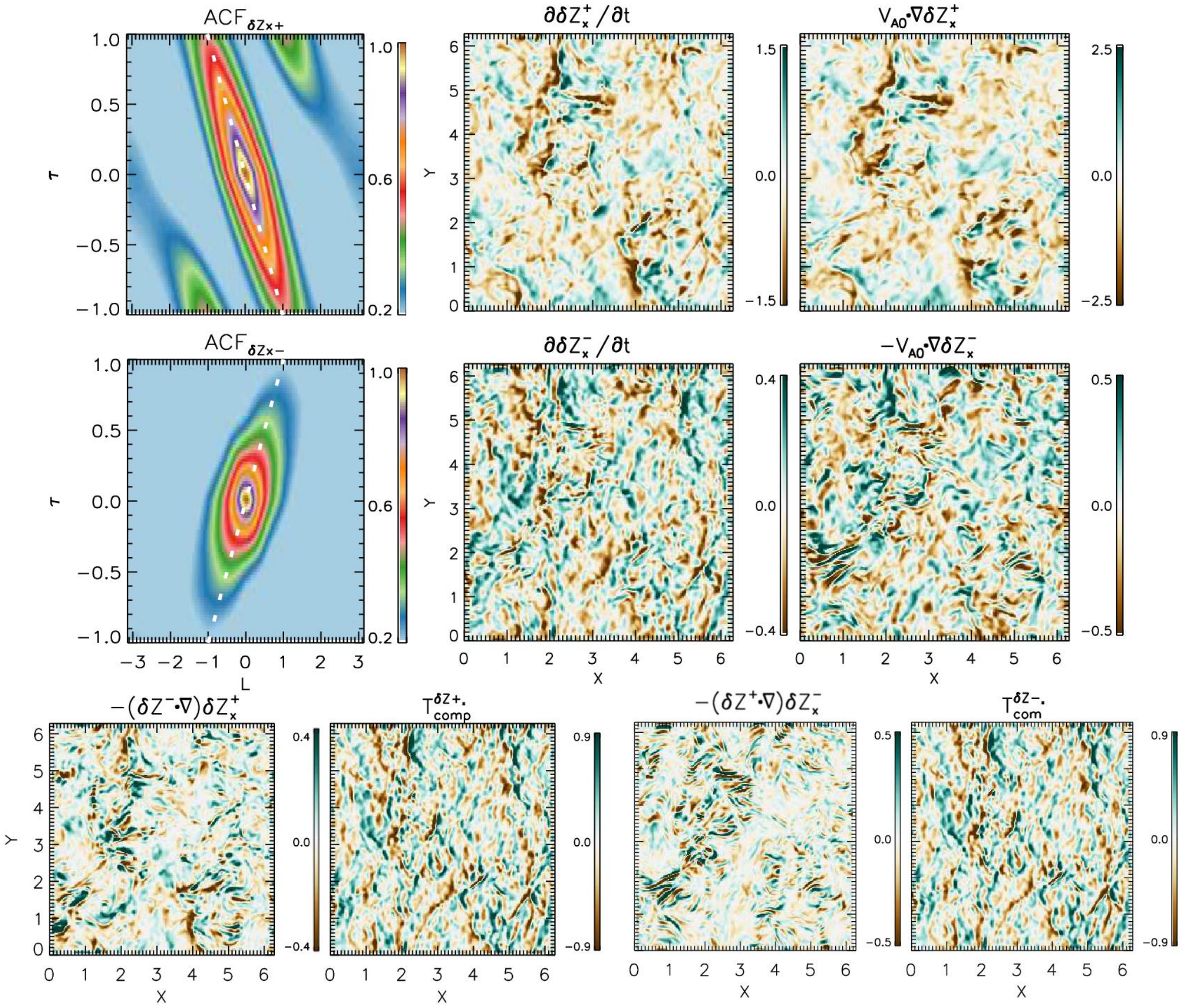

Supplementary Fig. 7 Distributions of ACFs and the terms for δZ_x^+ and δZ_x^- for the case with a small perturbation of $\delta B_{rms}/B_0 \approx 0.1$ and an initial cross-helicity of 0.7.

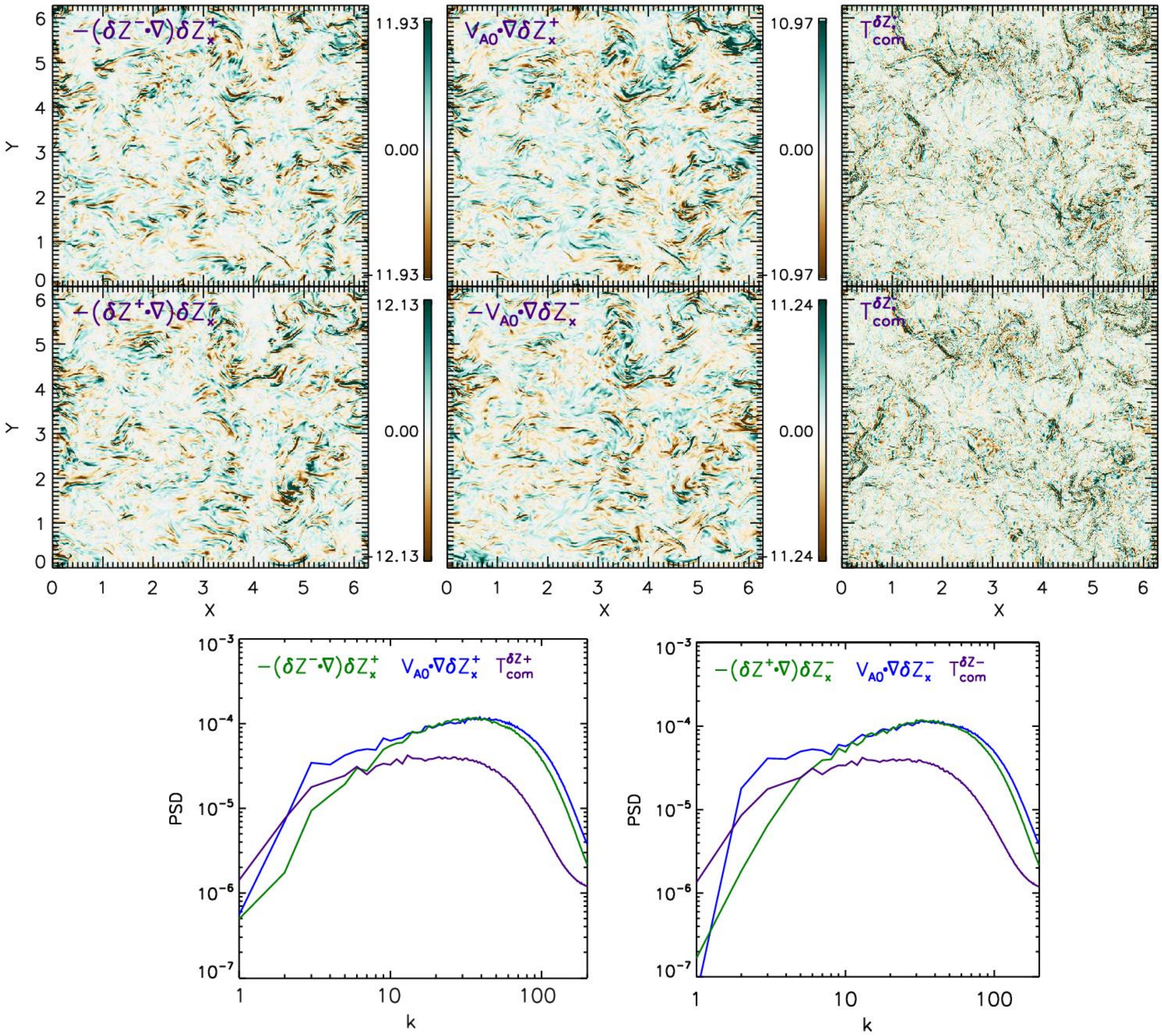

Supplementary Fig. 8 2D distributions of the discretized $\pm(\mathbf{V_{A0}} \cdot \boldsymbol{\nabla})\delta Z_x^{\pm}$, $-(\boldsymbol{\delta Z}^{\mp} \cdot \boldsymbol{\nabla})\delta Z_x^{\pm}$, $T_{\mathrm{com}}^{\delta Z_x^{\pm}}$, and their PSDs as functions of wavenumber k at time $= 4.0$ for the case with an initial cross-helicity of 0.0.

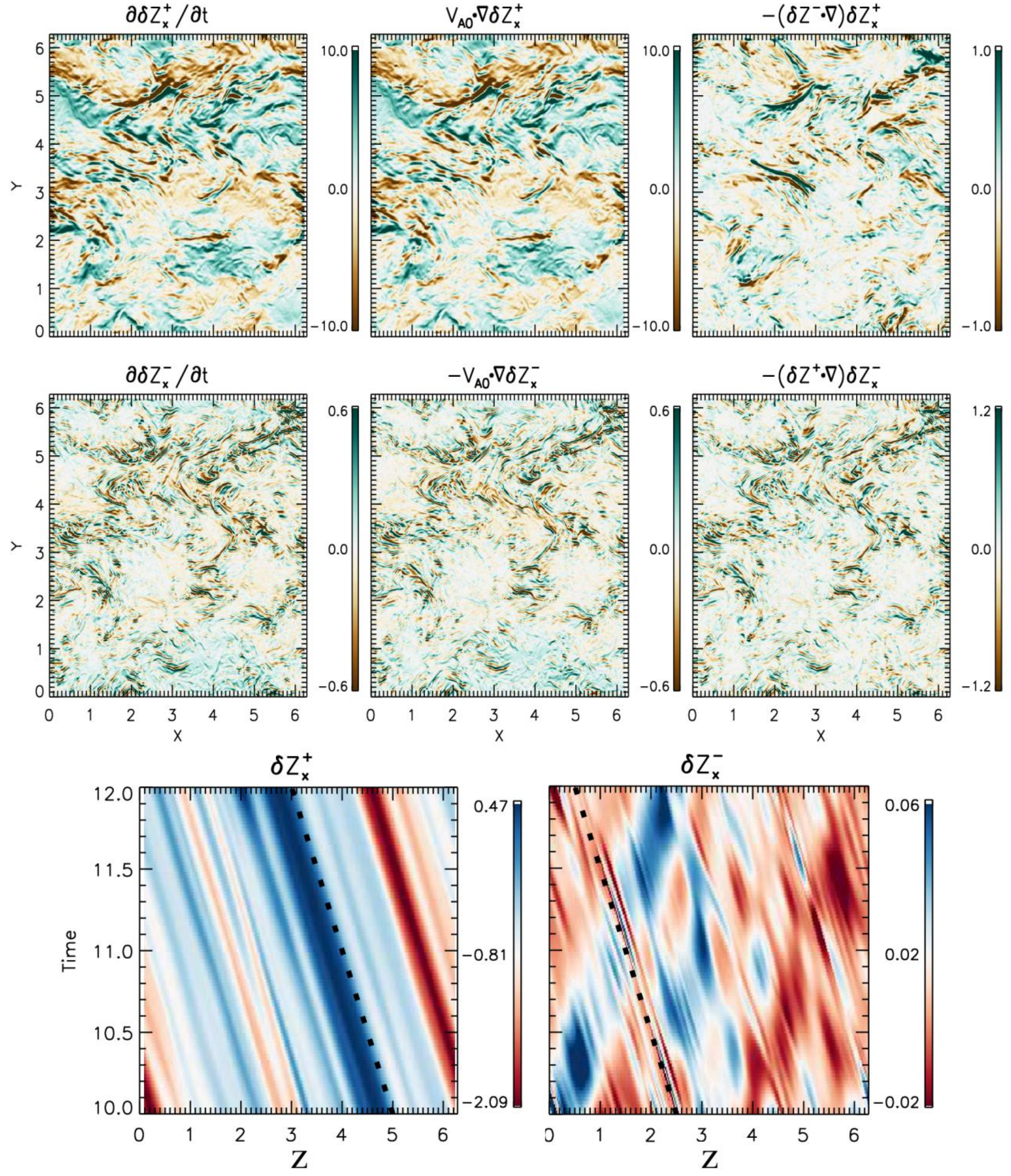

Supplementary Fig. 9 2D distributions of the discretized $\frac{\partial \delta Z_x^\pm}{\partial t}$, $\pm(\mathbf{V_{A0}} \cdot \boldsymbol{\nabla})\delta Z_x^\pm$, $-(\boldsymbol{\delta Z}^\mp \cdot \boldsymbol{\nabla})\delta Z_x^\pm$ term and time-distance (time$-z$) diagrams of δZ_x^+ and δZ_x^- for our imbalanced RMHD simulation.

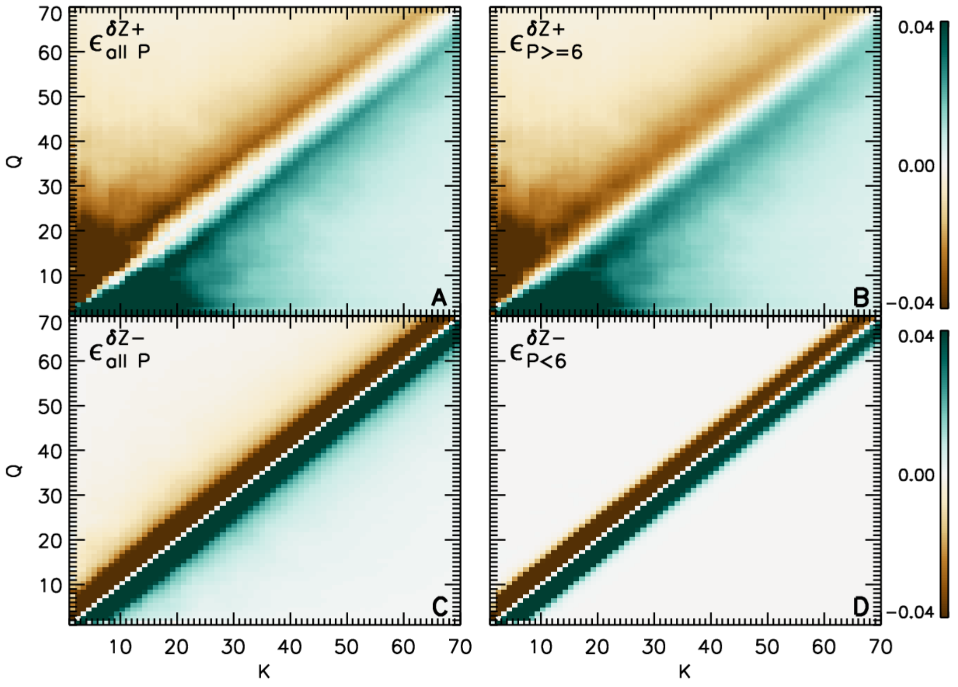

Supplementary Fig. 10 **(A** and **B**) Energy transfer rates from shell Q to shell K by mediation of all P modes (**A)** and by mediation of P modes with $P \geq 6$ for $\delta\mathbf{Z}^{+}$ (**B**). (**C** and **D**) Energy transfer rates from shell Q to shell K by mediation of all P modes **(C)** and by mediation of P modes with $P < 6$ for $\delta\mathbf{Z}^{-}$ (**D**) at time $= 4.0$ for the case with an initial cross-helicity of 0.7.

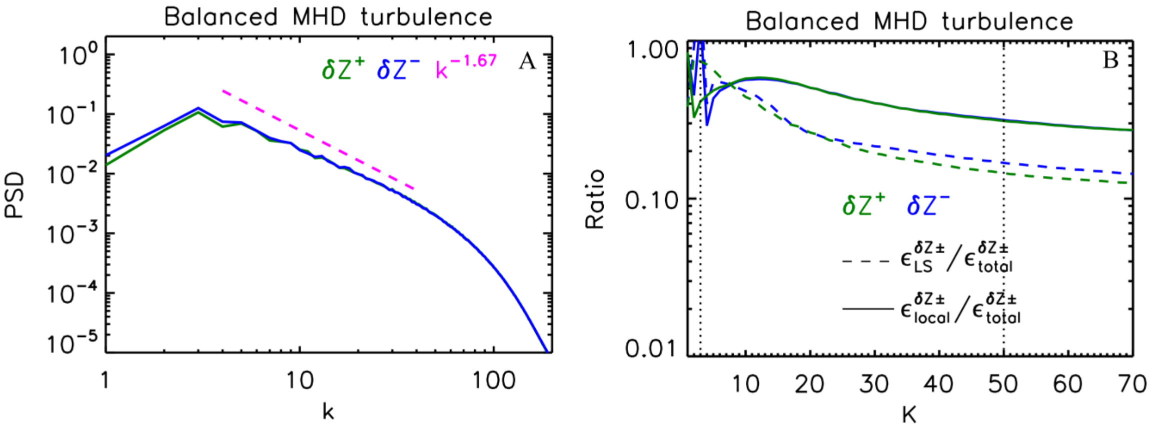

Supplementary Fig. 11 **(A)** Power spectra of the Elsässer variables $\delta\mathbf{Z}^{+}$ (green lines) and $\delta\mathbf{Z}^{-}$ (blue lines). (B) Ratios of the energy transfers from the large-scale interactions (dashed lines) and from the local interactions (solid lines) to the transfer due to all triadic interactions on a band of K-shells for $\delta\mathbf{Z}^{+}$(green lines) and $\delta\mathbf{Z}^{-}$ (blue lines) at time $= 4.0$ for the case with an initial cross-helicity of 0.0.

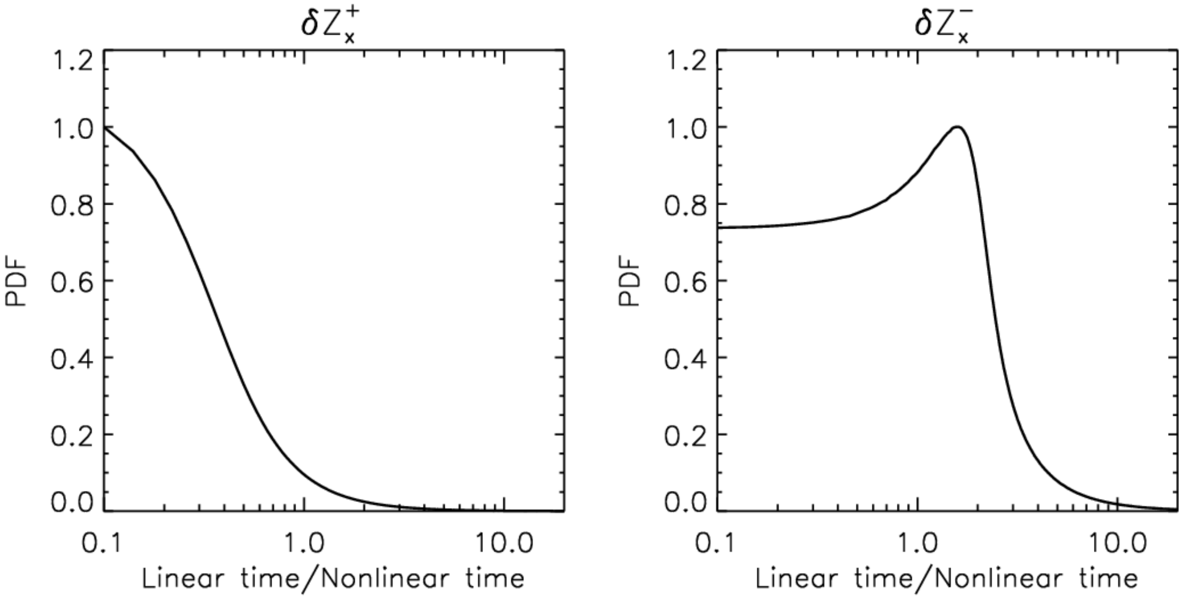

Supplementary Fig. 12 Probability density function (PDF) of linear time over nonlinear time for δZ_x^+ (left) and δZ_x^- (right) at $\mathrm{time} = 4.0$ for the case with an initial cross-helicity of 0.7. Here, the linear and nonlinear times are computed from the discretized terms $\left|\frac{\delta Z_x^{\pm}}{(\mathbf{V_{A0}}\cdot\nabla)\delta Z_x^{\pm}}\right|$ and $\left|\frac{\delta Z_x^{\pm}}{(\boldsymbol{\delta}\mathbf{Z}^{\mp}\cdot\nabla)\delta Z_x^{\pm}}\right|$, respectively.

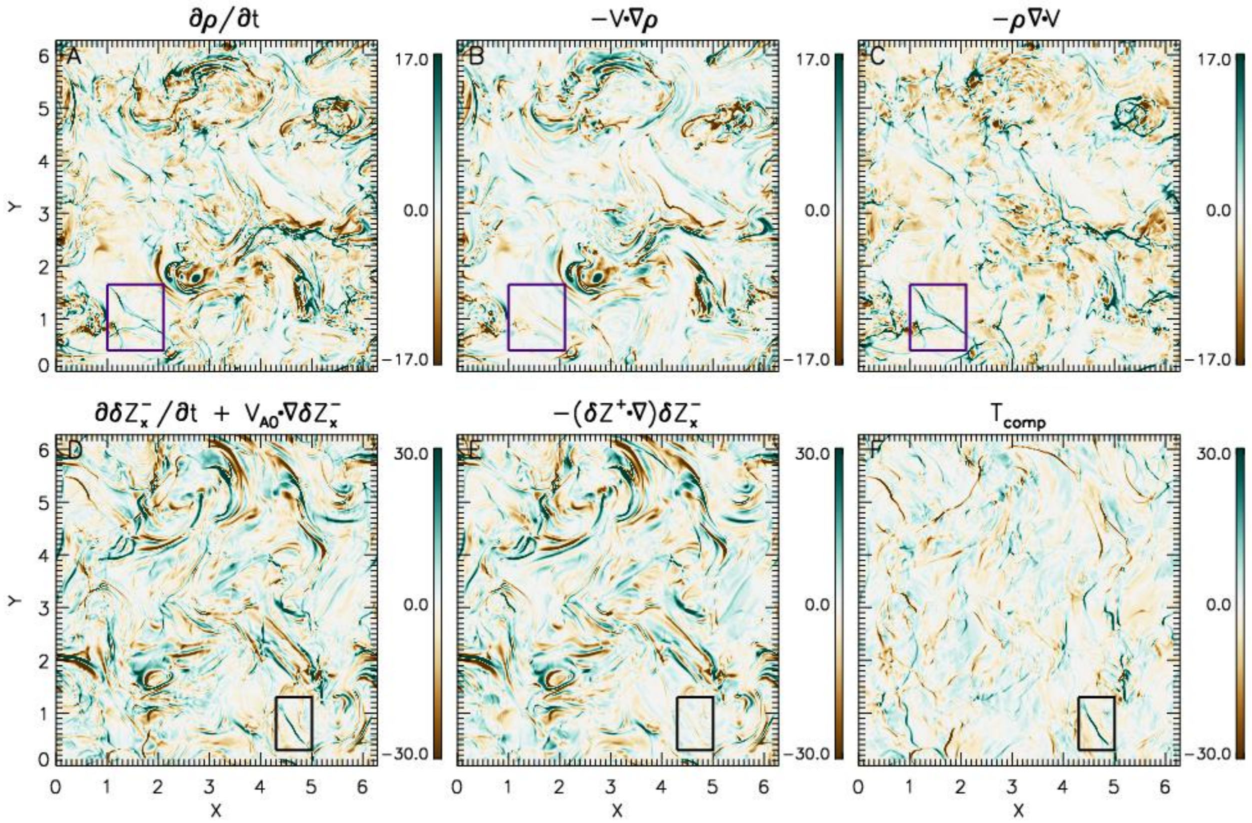

Supplementary Fig. 13 2D distributions of the discretized terms $\partial\rho/\partial t$ (**A**), $-\mathbf{V}\cdot\boldsymbol{\nabla}\rho$ (**B**), $-\rho\boldsymbol{\nabla}\cdot\mathbf{V}$ (**C**), $\frac{\partial \delta Z_x^-}{\partial t}$+$(\mathbf{V_{A0}}\cdot\boldsymbol{\nabla})\delta Z_x^-$ (**D**), $-(\delta\mathbf{Z}^+\cdot\boldsymbol{\nabla})\delta Z_x^-$ (**E**), and T_{comp} (**F**) at time $= 1.0$ for the case with an initial cross-helicity of 0.7. The purple boxes in the top row highlight the region where the Euler derivative of density with respect to time is predominantly controlled by the compressible term related to the divergence of the velocity field. The black boxes in the bottom row highlight the region where the "Lagrangian" derivative of δZ_x^- in the $\mathbf{V_{A0}}$ frame of motion is predominantly controlled by the corresponding compressible term.

Supplementary Reference

1. E. Marsch, A. Mangeney, Ideal MHD equations in terms of compressible Elsässer variables, *J. Geophys. Res.*, 92, 7363 (1987).
