# Supplementary material for: Energy transfer of imbalanced Alfvénic turbulence in the heliosphere
Source: Nat Commun. 2023 Dec 2;14:7955. doi: 10.1038/s41467-023-43273-4 (PMC10692179; doi:10.1038/s41467-023-43273-4)
Supplement: Supplementary file 2 — Description of Additional Supplementary Files [file 41467_2023_43273_MOESM2_ESM.pdf]

Description of Additional Supplementary Files

Supplementary Movie 1: Time evolution of δZ_x^+, δZ_x^-, $|\delta \mathbf{Z}^+|$, and $|\delta \mathbf{Z}^-|$ for the time from 4.0 until 6.0 for the case with an initial cross-helicity of 0.7.

Supplementary Movie 2: Time evolution of the evolution term $\frac{\partial \delta Z_x^\pm}{\partial t}$, the nonlinear term $-\left(\boldsymbol{\delta Z}^\mp \cdot \boldsymbol{\nabla}\right)\delta Z_x^\pm$, the linear term $\pm(\mathbf{V_{A0}} \cdot \boldsymbol{\nabla})\delta Z_x^\pm$, and the power spectra of $-\left(\boldsymbol{\delta Z}^\mp \cdot \boldsymbol{\nabla}\right)\delta Z_x^\pm$ and $\pm(\mathbf{V_{A0}} \cdot \boldsymbol{\nabla})\delta Z_x^\pm$ for the time from 4.0 until 6.0 for the case with an initial cross-helicity of 0.7.
